# Identification and validation of pyroptosis-related gene landscape in prognosis and immunotherapy of ovarian cancer

**DOI:** 10.1186/s13048-022-01065-2

**Published:** 2023-01-27

**Authors:** Lingling Gao, Feiquan Ying, Jing Cai, Minggang Peng, Man Xiao, Si Sun, Ya Zeng, Zhoufang Xiong, Liqiong Cai, Rui Gao, Zehua Wang

**Affiliations:** Department of Obstetrics and Gynecology, Union Hospital, Tongji Medical College, Huazhong University of Science and Technology, Wuhan, 430022 China

**Keywords:** Ovarian cancer, Pyroptosis, Tumor microenvironment, Immune and immunotherapy

## Abstract

**Background:**

Emerging evidence has highlighted the biological significance of pyroptosis in tumor tumorigenesis and progression. Nonetheless, the potential roles of pyroptosis in tumor immune microenvironment and target therapy of ovarian cancer (OC) remain unknown.

**Methods:**

In this study, with a series of bioinformatic and machine learning approaches, we comprehensively evaluated genetic alterations and transcriptome profiles of pyroptosis-associated genes (PYAGs) with TCGA-OV datasets. Consensus molecular clustering was performed to determine pyroptosis-associated clusters (PACs) and gene clusters in OC. Subsequently, component analysis algorithm (PCA) was employed to construct Pyrsig score and a highly accurate nomogram was established to evaluate its efficacy. Meanwhile, we systematically performed association analysis for these groups with prognosis, clinical features, TME cell-infiltrating characteristics, drug response and immunotherapeutic efficacy. Immunohistochemistry was conducted to verify molecular expression with clinical samples.

**Results:**

The somatic mutations and copy number variation (CNV) of 51 PYRGs in OC samples were clarified. Two distinct PACs (PAC1/2) and three gene clusters (A/B/C) were identified based on 1332 OC samples, PAC1 and gene cluster A were significantly associated with favorable overall survival (OS), clinicopathological features and TME cell-infiltrating characteristics. Subsequently, Pyrsig score was successfully established to demonstrate the prognostic value and immune characteristics of pyroptosis in OC, low Pyrsig score, characterized by activated immune cell infiltration, indicated prolonged OS, increased sensitivity of some chemotherapeutic drugs and enhanced efficacy of anti-PD-L1 immunotherapy, Consequently, a nomogram was successfully established to improve the clinical applicability and stability of Pyrsig score. With clinical OC samples, GSDMD and GZMB proteins were validated highly expressed in OC and associated with immune infiltration and Pyrsig score, GZMB and CD8 proteins were regarded as independent prognostic factors of OC.

**Conclusion:**

This work revealed pyroptosis played a non-negligible role in prognosis value, clinicopathological characteristics and tumor immune infiltration microenvironment in OC, which provided novel insights into identifying and characterizing landscape of tumor immune microenvironment, thereby guiding more effective prognostic evaluation and tailored immunotherapy strategies of OC.

**Supplementary Information:**

The online version contains supplementary material available at 10.1186/s13048-022-01065-2.

## Introduction

Ovarian cancer (OC) remains the most aggressive and fatal tumors in gynecological malignancy due to chemotherapy resistance and metastasis [1]. Over the past decades, infiltrated immune cells, as one of the most crucial components of the tumor microenvironment (TME) contexture, have been found to exert nonnegligible functions in tumorigenesis and metastasis of OC [2]. Immunotherapy represented by immune checkpoint inhibitors (ICIs) has been regarded as the prominent therapeutic strategies with its higher specificity, better outcome, and fewer side effects [3, 4]. However, only a small fraction of OC patients could respond to such immunotherapy [5]. Increasing studies have suggested that the TME plays a crucial role in immunotherapy responsibility [6]. Therefore, evaluating the immune infiltration characteristics of TME in OC provide novel approaches to developing effective immunotherapeutic strategies.

Pyroptosis, a form of lytic and pro-inflammatory programmed cell death, is characterized by gasdermin (GSDM) family proteins or caspases activation to induce membrane permeabilization, cell swelling, and release of intracellular inflammatory cytokines, such as IL-18, IL-1β, HMGB1, and ATP, accompanied by an intense inflammatory responsiveness and activation of immune infiltration cells [7, 8]. The dysregulation of pyroptosis exerted great impacts on tumor progression and prognostic prediction of various cancers, including OC [9]. Researchers showed that pyroptosis induced by α-NETA can inhibit progression of OC through increasing gasdermin D (GSDMD)/caspase-4 expression [10]. NOD-like receptor protein 1 (NLRP1) mediated pyroptosis caused by silencing lncRNA HOTTIP could inhibit the growth of OC [11]. However, the functions and underlying mechanisms of PYAGs in immune microenvironment of OC remain elusive.

The tumor microenvironment plays a crucial role in the tumorigenesis and progression of tumor cells, which consists of cancer cells, stromal cells (fibroblasts, endothelial cells and mesenchymal stem cells), immune and inflammatory cells (lymphocytes, macrophages and myeloid cells), extracellular matrix as well as secreted factors and their receptors, such as immune checkpoints (PD-1/L1 and CTLA4), chemokines, cytokines and growth factors [12]. The multifaceted interactions between tumor cells and surrounding immune components in TME can affect tumor development and progression and induce immune escape and tolerance by secreting various molecules. Nowadays, considerable evidence has demonstrated complex crosstalk between PYAGs and tumor immune microenvironment [13]. Researchers found that gasdermin C (GSDMC) was transcriptionally regulated by PD-L1 in the nucleus under the condition of hypoxia, and further eliminated by TAM-derived caspase-8, which in turn induced pyroptosis in breast cancer cells [14]. A lack of GSDMD hampered the ability of CD8^+^ T cells to destroy tumor cells [15]. Additionally, activation of gasdermin E (GSDME) could turn a “cold” tumor that was lack of immune response into a “hot” tumor that was controlled by the immune system [16]. However, the afore-mentioned researches were all restricted to one or two PYAGs and cell types due to technical limitations, while pyroptosis and immune response are processes involving multiple steps and molecules, acted in a highly united and cooperative manner. Alternatively, numerous transcriptomics and genomic profiles provided favorable access to comprehensively analysis the interactions between pyroptosis and immune regulation. Therefore, recognizing immune cell infiltration characteristics mediated by pyroptosis will contribute to understanding the underlying mechanism of OC progression and immunotherapy.

Herein, we comprehensively evaluated the association between pyroptosis subtypes and TME infiltrating immune characteristics by integrating the transcriptomic and genomic data of 1332 OC samples from TCGA-OV and five GEO (GSE140082, GSE63885, GSE32062, GSE26193 and GSE17260) datasets. Two distinct pyroptosis-associated clusters (PACs) were identified with consensus molecular clustering based on the PYAGs expression. Three gene clusters were classified based on differentially expressed genes (DEGs) between two PACs. Moreover, we established Pyrsig score to predict prognostic value and response to immunotherapy of OC. Finally, the expression of CD8, GSDMB and GZMB in OC samples and their correlation with immune infiltration were validated with immunohistochemistry.

## Materials and methods

### Data acquisition and preprocessing

Additional file 1: Fig. S1 shows the workflow of the present work. Publicly available gene expression data and complete clinical annotations of OC were retrospectively screened from the NCBI Gene Expression Omnibus (GEO) (https://www.ncbi.nlm.nih.gov/geo/) and The Cancer Genome Atlas (TCGA) (https://portal.gdc.cancer.gov/) databases, and the expression data of 88 normal human ovarian samples were obtained from GTEx database (https://xenabrowser.net/datapages/). In total, two RNA-sequencing (RNA-seq) datasets and five GEO OC cohorts (GSE140082, GSE63885, GSE32062, GSE26193 and GSE17260) were included for subsequent analysis. For TCGA-OV, gene expression (fragments per kilobase million, FPKM) values were transformed into transcripts per kilobase million (TPM) values to be identical with these from microarrays [17]. From the GEO microarray database, all the raw CEL files and clinical features were downloaded, and batch effects caused by non-biotechnological bias among different datasets were corrected by applying the “Combat” algorithm with ‘SVA’ R package [18]. The somatic mutation and copy number variation (CNV) profiles were downloaded from the Genomic Data Commons (GDC, https://portal.gdc.cancer.gov/). Somatic mutation data, which were calculated using R package ‘maftools’, were sorted in Mutation Annotation Format (MAF) format. Significant microduplication/deletion of copy number were explored using GISTIC 2.0 with a threshold of FDR Q < 0.05. Methylation analysis of 51 PYAGs was performed using DiseaseMeth 2.0 (http://bio-bigdata.hrbmu.edu.cn/diseasemeth/index.html), which provides a platform that integrates human DNA methylation information and metadata from publicly available datasets. The screening conditions were set as follows: Control Type: controls from the same tissue/cell line; Significant *p*-value < 0.05; Absolute Methylation Difference > 0.1. Patients without overall survival (OS) information, samples belong to borderline tumor, benign tissues or normal ovarian tissues and GEO samples belong to TCGA were excluded from this work, and a total of 1332 OC patients were included in the subsequent analyses (Additional file 9: Table S1 and S2).

### Consensus molecular clustering of PYAGs

A total of acknowledged 51 PYAGs were retrieved from the MSigDB Team (REACTOME_PYROPTOSIS) (http://www.broad.mit.edu/gsea/msigdb/) and published articles [19–21]. The full details of PYAGs were summarized in Additional file 9: Table S3. Consensus unsupervised clustering analysis was employed to identify distinct PACs based on the expression of PYAGs with R package “ConsensusClusterPlus”, which was conducted for 1000 times repetitions to ensure the stability of classification [22]. The criteria were performed as follows: the cumulative distribution function (CDF) curve with gradual and smooth acceleration; no groups with a small sample size; the increased intragroup correlation and decreased intergroup correlation.

### Gene set variation analysis (GSVA) and function annotation

To investigate the biological process between different PACs, gene set variation analysis (GSVA) was performed with “GSVA” R packages, and the hallmark.

Gene set (c2.cp.kegg.v7.4) were extracted from the MSigDB database for GSVA analysis [23, 24]. Adjusted *P* with value less than 0.05 was regarded as statistically significance. Functional annotation for PYAGs were performed by the clusterProfiler R package [25], with the cutoff value of FDR < 0.05.

### Estimation of TME immune cell infiltration and immune response predictor

The ssGSEA (single-sample gene-set enrichment analysis) algorithm were employed to evaluate the relative abundance of each type immune cell infiltration in the TME of OC [26], which was represented by an enrichment score in ssGSEA analysis and normalized to unity distribution from 0 to 1. The fractions of 22 distinct immune cell subsets of every OC sample were estimated by the CIBERSORT algorithm with the deconvolution approach [27]. ESTIMATE (Estimation of Stromal and Immune Cells in Malignant Tumors using Expression Data) algorithm, which concludes the tumor cellularity as well as the tumor purity according to the unique characteristics of the transcriptional profiles, was used to calculate the stromal score, immune score and ESTIMATE score, predicting the level of infiltrating immune and tumor purity [28]. A high immune score along with low tumor purity indicated abundant immune cell infiltration in tumor tissues. We also utilized Microenvironment Cell Populations-counter (MCP-counter), TIMER, QUANTISEQ, EPIC and XCELL to delineate the immunogenomic landscape of immune infiltration and functions in ovarian cancer [29–32].

### Identification of DEGs between PACs and their functional annotation

Two distinct PACs in patients were concluded by above consensus clustering algorithm. DEGs associated with different PACs were identified using R package “limma”. Adjusted *P* value less than 0.001 was set as significance filtering criteria. To further investigate potential biological functions of DEGs and identify the related gene functions and enriched pathways, package “clusterprofiler” was conducted by software R [25].

### Relationship of PACs with clinical characteristics and prognosis of OC

To explore the clinical significance of the different subtypes identified by consensus clustering algorithm, relationships between molecular subtypes and clinicopathological characteristics, prognosis in OC patients were analyzed. The patient characteristics included age, differentiation grade, and FIGO stage. Moreover, differences in OS among different clusters were evaluated with Kaplan–Meier curves generated by the R packages “survival” and “survminer”.

### Construction of Pyrsig scoring system

To quantify pyroptosis patterns of individual tumors, a scoring system was developed based on pyroptosis-associated prognostic genes, termed as Pyrsig score. Specifically, overlapping DEGs were employed to perform prognostic analysis for each gene using a univariate Cox regression model. Then patients were classified into three different gene clusters (gene cluster A/B/C) for deeper analysis using consensus clustering algorithm based on PYAGs with prognostic value. Concomitantly, PCA was employed to establish Pyrsig score based on the expression profile of 889 genes with survival significance, and principal components 1 and 2 were extracted and served as the final gene signature scores. The Pyrsig score was calculated as follows:

$$\mathrm{Pyrsig}\;\mathrm{score}=\sum\mathrm(\mathrm{PC}1i+\mathrm{PC}2i\mathrm)$$

where i is the expression of prognostic genes in ovarian cancer.

### Correlation between Pyrsig score and drug susceptibility, immunotherapy

Genomics of Drug Sensitivity in Cancer (GDSC, https://www.cancerrxgene.org/) [33], as the largest public pharmacogenomics databases, was used to predict chemotherapy drug sensitivity. The semi-inhibitory concentration (IC50) values of chemotherapeutic drugs commonly for OC treatment were estimated using the package “pRRophetic” [34].

We performed a systematical search for immune checkpoint genes, immune chemokines, interleukin, interferon as well as epithelial-mesenchymal transition (EMT) -related genes, which were extracted to explore their relationship with Pyrsig score [35]. Immunophenoscore (IPS) could evaluate responsiveness to anti-PD-1 immunotherapy [26]. The Tumor Immune Dysfunction and Exclusion (TIDE) algorithm was utilized to explore distinct tumor immune escape mechanisms [36], including dysfunction of tumor infiltration cytotoxic T lymphocytes (CTLs) and exclusion of CTLs by immunosuppressive factors. Furthermore, the immunotherapeutic cohorts, advanced urothelial cancer with intervention of atezolizumab, an anti-PD-L1 antibody (IMvigor210 cohort) [37] (http://research-pub.Gene.com/imvigor210corebiologies), was also screened to evaluate the response to immunotherapy.

### Immunohistochemistry staining

65 paraffin-embedded ovarian cancer tissue microarrays were obtained from Department of Obstetrics and Gynecology, Union Hospital, Tongji Medical College, Huazhong University of Science and Technology. All participants were informed consent and approved by the Research Ethics Committee of Union Hospital. Immunohistochemical staining procedure was performed by the streptavidin-peroxidase (SP) kit (ZSGB-BIO, China) based on the manufacturer’s instructions. Briefly, the paraffin sections were deparaffinized and rehydrated, followed by antigen retrieval with Tris-EDTA buffer (pH 6.0) through microwave treatment. Endogenous peroxidase activity was blocked with 10% hydrogen peroxide for 30 min, and nonspecific binding was blocked with normal goat serum for 30 min at room temperature. The tumor sections were then incubated with primary antibodies overnight at 4 °C. The working concentrations of primary antibodies were as follows: Rabbit monoclonal anti-CD8 (1:100, Abcam Cambridge, ab237709), Rabbit polyclonal anti-GSDMD (1:200, Abclonal China, A18281) and Rabbit polyclonal anti-GZMB (1:200, Abclonal China, A2557), respectively. Phosphatebuffered saline (PBS) served as negative control instead of primary antibody, rabbit IgG served as isotype control instead of primary antibody. The slides were then labelled with biotinylated anti-rabbit IgG and peroxidase-labeled streptavidin. After washed with PBS for three times, the sections were stained with DAB and observed under a microscope. Based on the chromatosis intensity, no pigmentation, light yellow, brown yellow, and dark brown were scored 0, 1, 2, and 3, respectively. The percentage of stained cells observed in the visual field, less than 5%: 0, 5–25%: 1, 26–50%: 2, 51–75%: 3, and greater than 75%: 4, respectively. The final score was obtained by multiplying above two scores: 0–2, (−); 3–4, (+); 5–8, (++); and 9–12, (+++). To control errors, the stained images were independently observed by two senior pathologists.

### Statistical analysis

R software (version 4.1.1) was employed for all statistical analyses. The statistical significance between two groups was calculated by Student’s t tests or Wilcoxon tests, as appropriate. Comparisons among more than two groups estimated by one-way ANOVA or Kruskal-Wallis tests. The “multcomp” package and “ggplot2” were employed to account for multiple testing. The chi-square test was applied to analyze the differences in clinicopathological parameters between Pyrsig score groups. The prognostic survival curves were achieved by the Kaplan–Meier plotter, and survival differences were evaluated with the log-rank test using R package “survminer”. Spearman analysis was conducted to calculate the correlation coefficient. A potential immune therapy response was predicted with TIDE algorithm. Two-sided with statistical significance was set at *P* < 0.05.

## Results

### Landscape of genetic characteristics and transcriptional patterns of pyroptosis-associated genes (PYAGs) in ovarian cancer

**Fig. 1:**
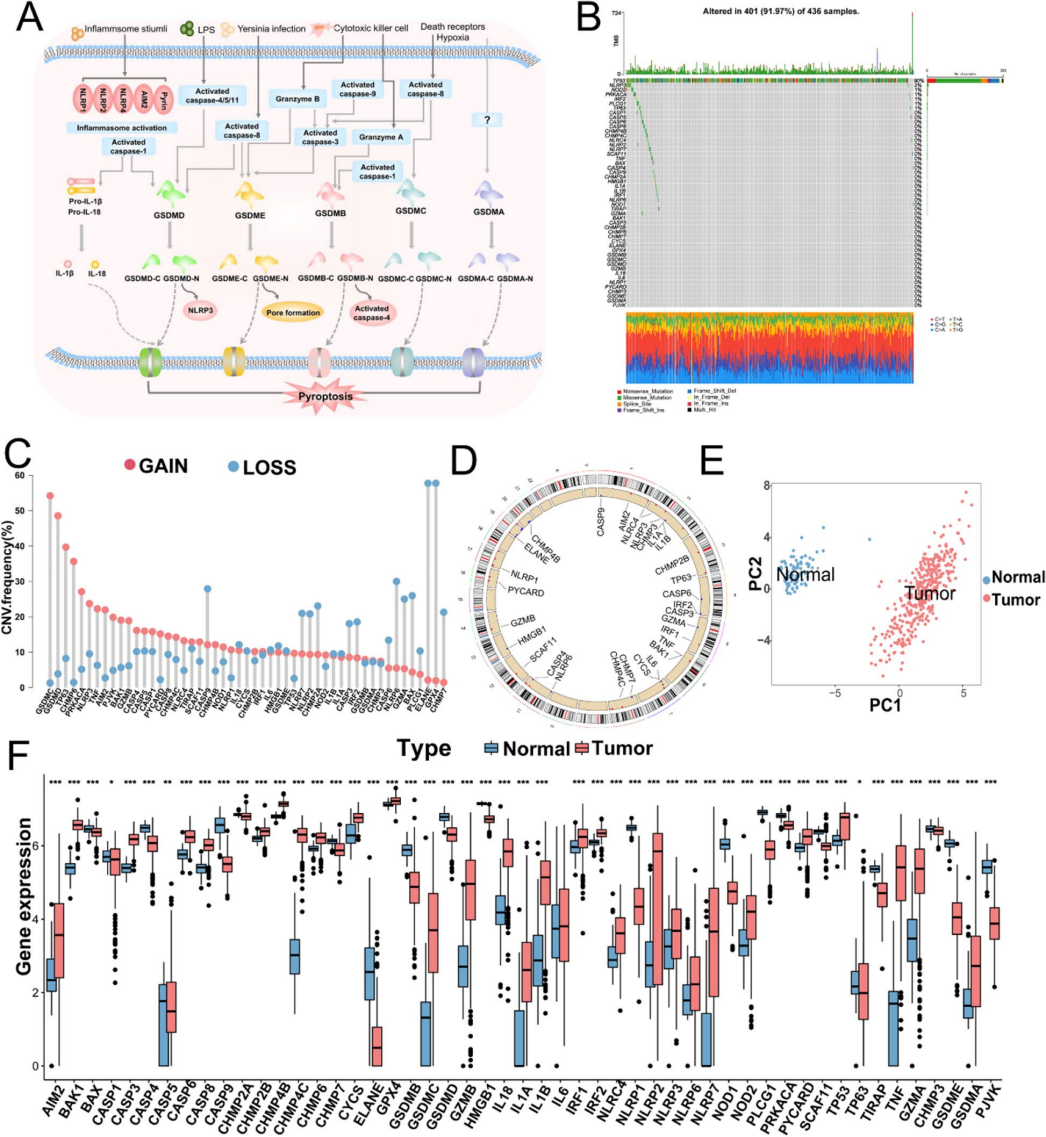
Landscape of genetic alterations and transcriptional patterns of PYAGs in ovarian cancer.** A** Summary of the potential biological functions of pyroptosis and their molecular regulation mechanism. **B** Genetic alterations of 51 PYAGs in 436 patients with ovarian cancer from TCGA-OV cohort. **C** CNV variation frequency of 51 PYAGs in ovarian cancer. Red dot represents the amplification frequency; blue dot represents the deletion frequency. **D** The location of CNV alteration of 51 PYAGs on 23 chromosomes. **E** Principal component analysis (PCA) for the expression of 51 PYAGs to distinguish ovarian cancer from normal samples in TCGA-OV cohort and GTEx data. Red dot: ovarian cancer samples; Blue dot: normal ovarian samples. **F** Expression levels of 51 PYAGs between ovarian cancer and normal ovarian tissues from TCGA-OV and GTEx cohorts. *P < 0.05; **P < 0.01; ***P < 0.001. PYAG: pyroptosis-associated genes; OV: ovarian cancer; TMB: tumor mutation burden; CNV: copy number variation; PCA: Principal component analysis

A total of 51 pyroptosis-associated genes (PYAGs) were finally identified and included in this study. Their potential biological functions regarding pyroptosis and molecular regulation mechanisms are summarized in Fig. 1A. We show somatic mutations and copy number variation (CNV) of these 51 PYAGs in 436 ovarian cancers from the TCGA-OV database with a waterfall diagram. A relatively high mutation frequency observed in this TCGA-OV cohort (Fig. 1B). A total of 400 (91.97%) among 436 samples experienced mutations in PYAGs (Fig. 1B). TP53 (88%) exhibited the highest mutation frequency, followed by NLRP3 (3%), NLRP2 (3%) NOD1 (2%), NLRP6 (1%), GSDMB (1%), NLRC4 (1%), NOD2 (1%), TP63 (1%), CASP5 (1%), IRF1/2 (all 1%), NLRP1/7 (all 1%), PLCG1(1%), CASP1/6/8 (all 1%), CHMP4C (1%), PPKACA (1%), and TNF (1%), while the other PYAGs did not show any mutations in these OC samples (Fig. 1B). As TP53 showed the highest mutation frequency, we explored the relationship between TP53 mutation and PYAGs expression. The results showed that expression levels of 2 (NLRC4 and IL1A) among 51 PYAGs were significantly associated with TP53 mutation status (Additional file 2: Fig. S2A-B). As to CNV in PYAGs, GSDMD, GSDMC, TP63, CHMP6, PRKACA, NLRP3, TNF, AIM2, PJVK, BAK1, GZMB, CASP4, CASP5, CASP1, PYCARD, CASP8, CHMP4C, NLRC4, CHMP4B, and CHMP2B showed widespread CNV amplifications, while CASP9, NLRP7, NLRP2, CHMP2A, CASP3, IRF2, CASP6, NLRP6, GZMA, BAX, PLCG1, ELANE, GPX4, and CHMP7 showed prevalent CNV deletions (Fig. 1C). The locations of CNV alterations of 51 PYAGs on their respective chromosomes were shown in Fig. 1D. Furthermore, principal component analysis (PCA) based on expression of these 51 PYAGs in TCGA-OV and GTEx samples revealed that PYAGs could completely distinguish OV samples from normal samples (Fig. 1E-F). We further analyzed DNA methylation levels of 51 PYAGs with DiseaseMeth 2.0, the results showed AIM2, CASP1, CASP8, GSDMC and NLRP6 exhibited significantly lower methylation levels compared with those in normal control group (Additional file 2: Fig. S2C-G and Additional file 9: Table S4).

We further investigated mRNA expression levels of PYAGs between OC and normal samples in TCGA-OV and GTEx database, and found a positive correlation between mRNA expression and CNV alterations in most of PYAGs. Compared to normal ovarian tissues, the mRNA expression levels of PYAGs with CNV gain was markedly increased in OC tissues, such as GSDMC, CHMP6, NLRP3, TNF and AIM2, while PYAGs with CNV loss showed lower expression in tumors, such as CASP9, CHMP7, ELANE, PLCG1 and BAX. However, some PYAGs with CNV gain, such as GSDMD, TP63, PRKACA, PJVK and CASP4, showed downregulated mRNA expression in ovarian cancers, and some PYAGs with CNV loss, such as IL18, GPX4, GZMA, NLRP6 and CASP6, showed upregulated mRNA expression in ovarian cancers (Fig. 1F). Thus, while CNV can explain many observed changes in PYAGs expression, CNV is not the only factor involved in the regulation of mRNA expression. The above findings indicate the high heterogeneity of genomic and transcriptomic alteration landscape in PYAGs of OC patients, suggesting that pyroptosis might play a crucial role in OC development and progression.

### Identification of pyroptosis-associated subtypes in OC

**Fig. 2:**
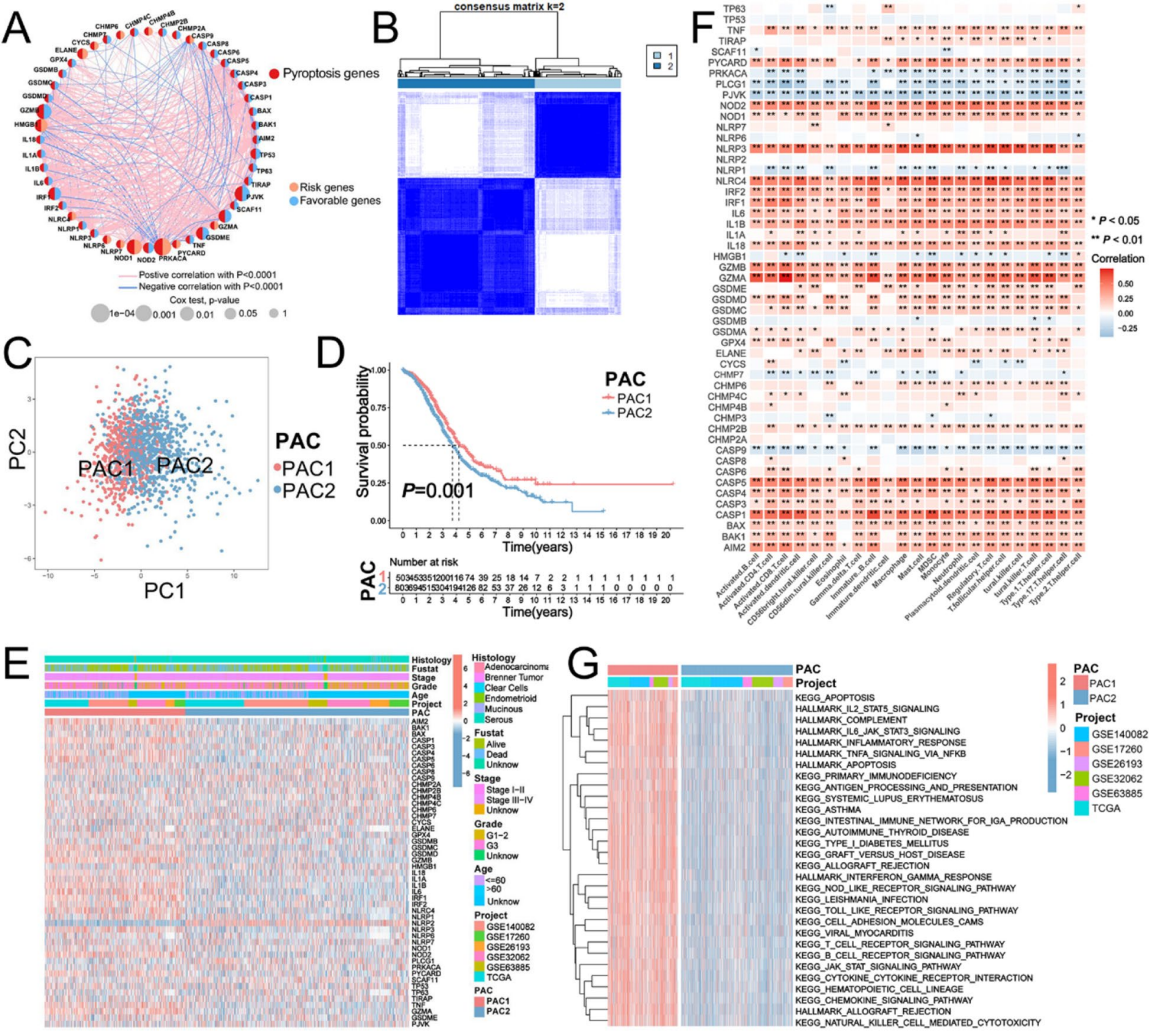
Consensus clustering to identify PACs and their correlation with clinicopathological and biological characteristics.** A** Interaction among PYAGs in ovarian cancer shown by network. **B** Two PACs (k = 2) and their correlation areas calculated by consensus clustering algorithm. **C** Differences in the transcription profiles between the two PACs analyzed by PCA. **D** Survival difference between the two PACs shown by Kaplan-Meier curves based on five GEO cohorts (GSE140082, GSE17260, GSE26193, GSE32062, and GSE63885) and TCGA-OV cohort. **E** Differences in clinicopathologic features, expression levels of PYAGs between two PACs in OC cohorts with heatmap. **F** Correlation between 51 PYAGs and immune cells in the gathered TCGA-OV cohort by heatmap. **G** Activated biological pathways in two PACs visualized by heatmap through GSVA enrichment analysis. *P < 0.05; **P < 0.01; ***P < 0.001. PYAG: pyroptosis-associated genes; PAC: pyroptosis-associated cluster; OV: ovarian cancer; PCA: Principal component analysis; GSVA: Gene Set Variation Analysis; OS: overall survival

Firstly, we explored prognostic value of PYAGs in patients across 33 cancer types. We found that all PYAGs were associated with overall survival of patients in at least one cancer type (Additional file 2: Fig. S2H). Subsequently, a total of 1332 ovarian cancer samples from six eligible OC cohorts (TCGA-OV, GSE140082, GSE63885, GSE32062, GSE26193, and GSE17260) were enrolled for further analysis. Kaplan–Meier analysis and univariate Cox regression revealed the prognostic values of 49 PYAGs in these OC patients, and *P* < 0.05 was selected as the threshold for filtering (Additional file 2: Fig. S2I and Additional file 9: Table S5). The comprehensive landscape of PYAGs interactions, molecular connections, and their prognostic significance in patients with OC was demonstrated in a pyroptosis network (Fig. 2A and Additional file 9: Table S6). Furthermore, through Spearman’s correlation analysis, we found a strong relationship of the 51 PYAGs with the TME-infiltrating immune cells utilizing the GSEA algorithm in TCGA-OV database. Most of these PYAGs were positively correlated with the immune cells in varying degrees. However, six PYAGs (CASP9, CHMP7, NLRP1, PJVK, PLCG1 and PRKACA) was negatively related to almost all these immune infiltration cells (Fig. 2F). The above results indicated that cross-talk among different PYAGs may play critical roles in the formation of different pyroptosis patterns and TME cell-infiltrating characterization between individual tumors.

To further explore the involvement of pyroptosis in OC, a consensus clustering algorithm was employed to stratify the patients based on the qualitatively different expression of these 49 genes (Additional file 3: Fig. S3A-H). Accordingly, *k* = 2 was identified as an optimal selection for clarifying the entire samples into two pyroptosis-associated clusters (PAC) including PAC1 (*n* = 515 cases) and PAC2 (*n* = 819 cases) (Fig. 2B). Principal component analysis (PCA) analysis revealed significant differences in the pyroptosis transcription profiles between the two distinct clusters (Fig. 2C), and patients in PAC1 showed significant survival advantage compared with those with PAC2 (*P* = 0.001, Fig. 2D). Furthermore, as shown in Fig. 2E, Patients in PAC2 were preferentially related to advanced stage (FIGO III-IV) and poor differentiation (histological grade 3) compared to patients in PAC1 (*P* < 0.05).

### TME infiltration characteristics in distinct PACs

**Fig. 3:**
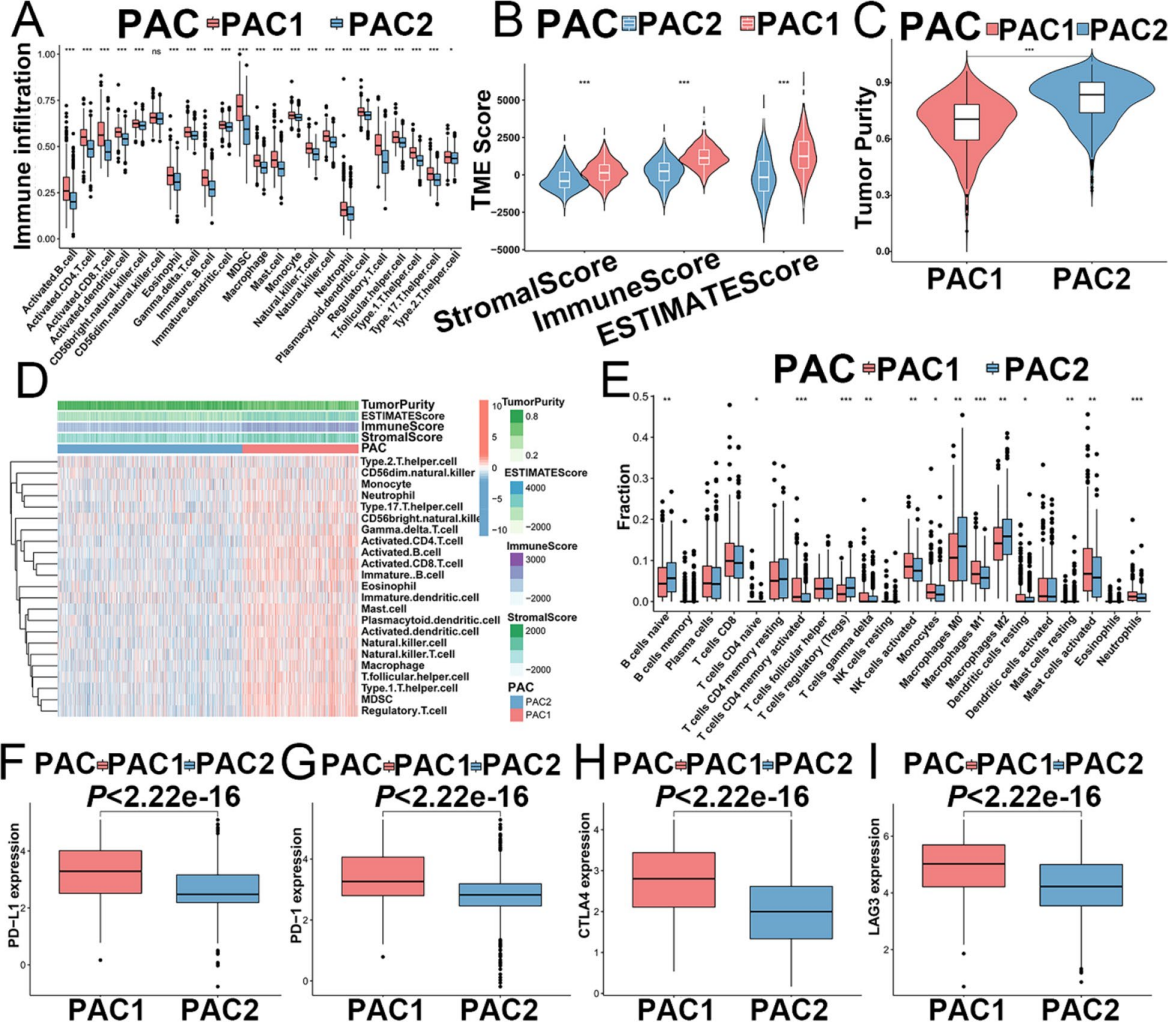
Characterization of TME infiltration characteristics in the two PACs.** A** Abundance of 23 tumor-infiltrating immune cells in two PACs using the ESTIMATE algorithm (Kruskal-Wallis H test). **B**,**C** TME score and tumor purity of different PACs analyzed with vioplot. **D** Correlation between TME score and tumor-infiltrating immune cells of two PACs by pheatmap. **E** Fraction of tumor-infiltrating lymphocyte cells in two PACs with CIBERSORT algorithm. **F**-**I** Expression levels of PD-L1, PD-1, CTLA4 and LAG3 between two PACs. *P < 0.05; **P < 0.01; ***P < 0.001. PYAG: pyroptosis-associated gene; TME: tumor microenvironment; ESTIMATE: Estimation of Stromal and Immune Cells in Malignant Tumors using Expression Data

To explore the biological behaviors related to the two distinct PACs, we conducted GSVA enrichment analysis against the Hallmarker gene set. As shown in Fig. 2G and Additional file 9: Table S7, PAC1 was markedly enriched in immune fully-activated pathways, including inflammatory response, interferon gamma response, natural killer cell mediated cytotoxicity, T cell and B cell receptor signaling pathway as well as NOD-like and Toll-like receptor signaling pathways, cell adhesion and JAK-STAT signaling pathways (Fig. 2G). While PAC2 was prominently associated with immune suppression biological process (Fig. 2G). Accordingly, subsequent analysis of TME immune cell infiltration with ssGSEA indicated that PAC1 showed higher level of infiltration of most immune cells than PAC2, including activated CD4^+^/CD8^+^T cells, activated B cells, natural killer cell, macrophage, eosinophil, mast cell and plasmacytoid dendritic cell (Fig. 3A). What’s more, we utilized ESTIMATE algorithm to evaluate immune infiltration (stromal score, immune score, and ESTIMATE score) and tumor cell purity (Tumor Purity) (Additional file 9: Table S8) of the tumors with PAC1 versus those with PAC2, which further demonstrated that PAC1 displayed higher immune scores, stromal score and ESTIMATE score compared with PAC2, and PAC2 showed a higher tumor purity than PAC1 (Fig. 3B-D), suggesting that abundant nontumor components (e.g., immune cells and stromal cells) were existed in ovarian cancer with PAC1. We also characterized the infiltration of 22 human immune cells in OC between the two clusters with CIBERSORT, which demonstrated obviously higher infiltration of activated memory CD4^+^ T cells, activated NK cells, M1 macrophages, T gamma delta cells, activated mast cells and neutrophils in the PAC1 compared to PAC2, while M0 and M2 macrophages, naive B cells, and regulatory T cells (Tregs) were significantly enriched in the PAC2 (Fig. 3E). Furthermore, the expression of immune checkpoints, such as PD-L1, PD-1, CTLA4 and LAG3, were significantly upregulated in PAC1 than those in PAC2 (Fig. 3F-I). These findings suggest that the two clusters had significantly distinct TME cell infiltration characterization.

### Gene subtypes based on pyroptosis phenotype-related DEGs in OC

**Fig. 4:**
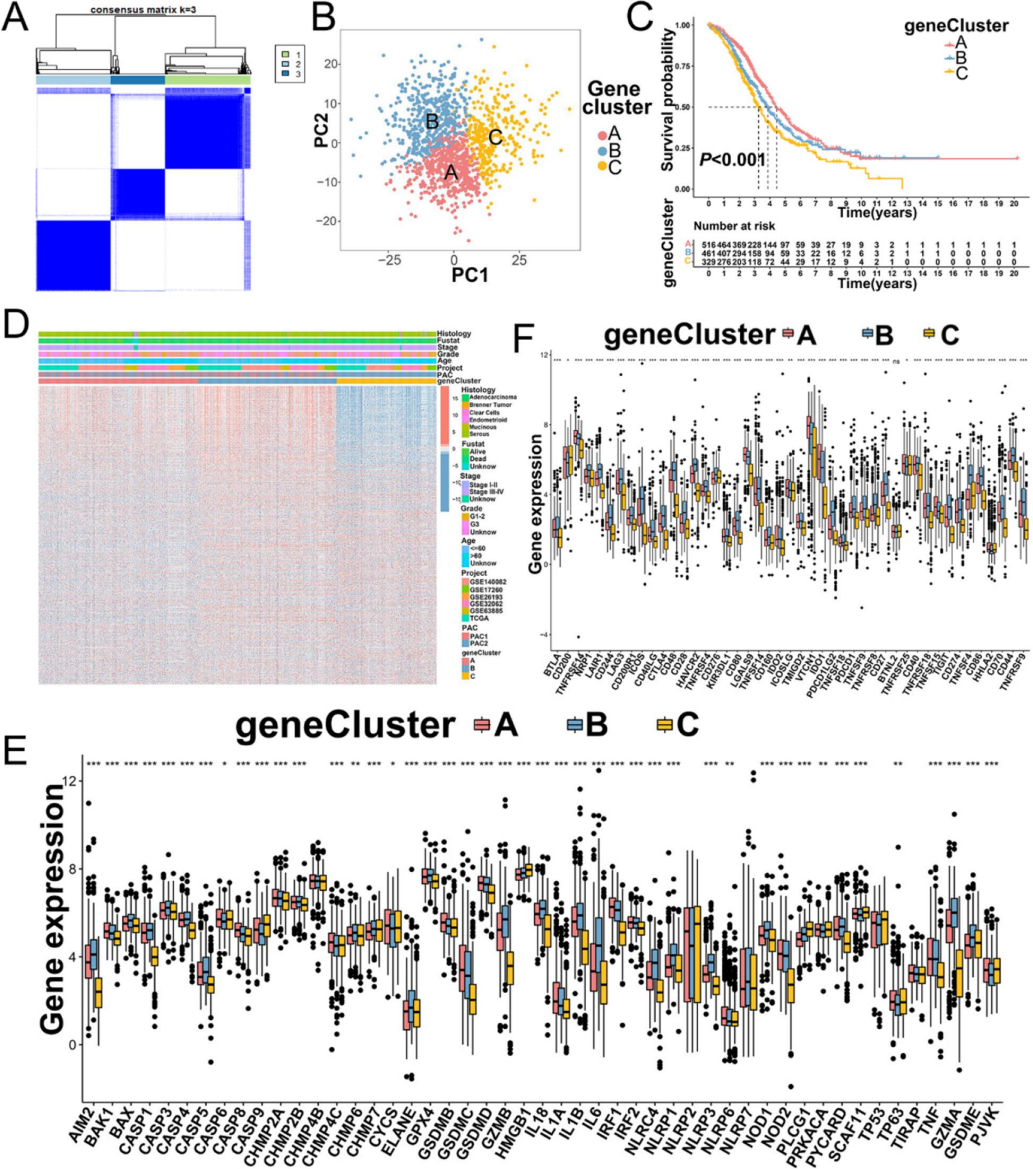
Construction of gene clusters based on PACs-related DEGs.** A** Identification of three gene clusters (*k* = 3) based on PACs-related DEGs by consensus clustering algorithm, gene cluster A (534 patients), gene cluster B (465 patients), and gene cluster C (335 patients). **B** The remarkable difference among transcriptome profiles of three gene clusters with PCA. **C** The OS of three gene clusters in TCGA and five GEO cohorts shown by Kaplan-Meier curves (log-rank tests, *P* < 0.001). **D** Relationships between clinicopathologic features and three gene clusters analyzed by unsupervised clustering. **E** Differences in the expression of 49 PYAGs among three gene clusters. **F** Differences in the expression levels of immune checkpoints among three gene clusters in TCGA and five GEO cohorts. *P < 0.05; **P < 0.01; ***P < 0.001. DEGs: Differentially expressed genes; PCA: Principal component analysis; OS: Overall survival

Although the PYAGs were classified into two clusters by consensus clustering algorithm in OC patients, the underlying genetic alterations and potential biological behavior within these clusters remains to be clarified. A total of 4342 overlapping DEGs were identified between two PACs. GO enrichment analysis showed that these DEGs were significantly enriched in biological processes related to immune regulation, such as T cell activation and leukocyte cell − cell adhesion (Additional file 3: Fig. S3I-K and Additional file 9: Table S9). KEGG analysis demonstrated DEGs were enriched in cytokine − cytokine receptor and tumor-related pathways (Additional file 3: Fig. S3L-N and Additional file 9: Table S9), suggesting that pyroptosis exerts a nonnegligible function in the immune regulation of the TME. We then employed Univariate Cox regression analysis to screen out 889 DEGs with significant favorable overall survival (OS) of OC patients (all *P* < 0.05) (Additional file 9: Table S10). Based on the transcriptional levels of 889 pyroptosis-related gene signatures, unsupervised consensus clustering algorithm was performed to obtain three distinct pyroptosis gene subtypes which were identified as gene cluster A (534 cases), gene luster B (465 cases), and gene cluster C (335 cases), respectively (Fig. 4A and Additional file 4: Fig. S4A-H). PCA analysis confirmed discernible dimensions among the transcription profiles of the three gene clusters (Fig. 4B). Survival analysis showed that patients in gene cluster C (335 patients) showed the worst survival outcome among the three subtypes (*P* < 0.001) (Fig. 4C). Furthermore, gene cluster C was associated with advanced FIGO stage and grade 3, and patients in PAC1 and gene cluster A showed favorable overall survival (Fig. 4D), which indicated that three distinct gene clusters were characterized by different clinicopathologic feature and survival outcome. Moreover, three gene clusters displayed significant differences in the expression of 49 PYAGs (Fig. 4E).

To explore the potential role of the pyroptosis-related gene signatures in the TME immune infiltration, we analyzed the expression of immune checkpoints, chemokine, cytokines and other factors among three gene clusters of OC, and found that the expressions of immune checkpoints (PD-L1, CTLA-4, and LAG3), chemokines (CXCL10, CCL5, and CXCL13), interleukins interferons (IFNG, IFNB1, and IFNAR2) and MHC molecules (HLA-A, HLA-B, and HLA-C) [26] were significantly upregulated in gene cluster A and B compared with those in gene cluster C (Fig. 4F and Additional file 4: Fig. S4I-L), suggesting that gene cluster A and B was regarded as immune activated characteristic. However, gene cluster C showed higher expression of molecules (TGF-β2 and Smad9) related with TGF-β/EMT pathway than gene cluster A and B (Additional file 4: Fig. S4M), indicating gene cluster C was deemed as stromal activated characteristic and tumor promotion.

### Construction of Pyrsig score and exploration of its clinical relevance

**Fig. 5:**
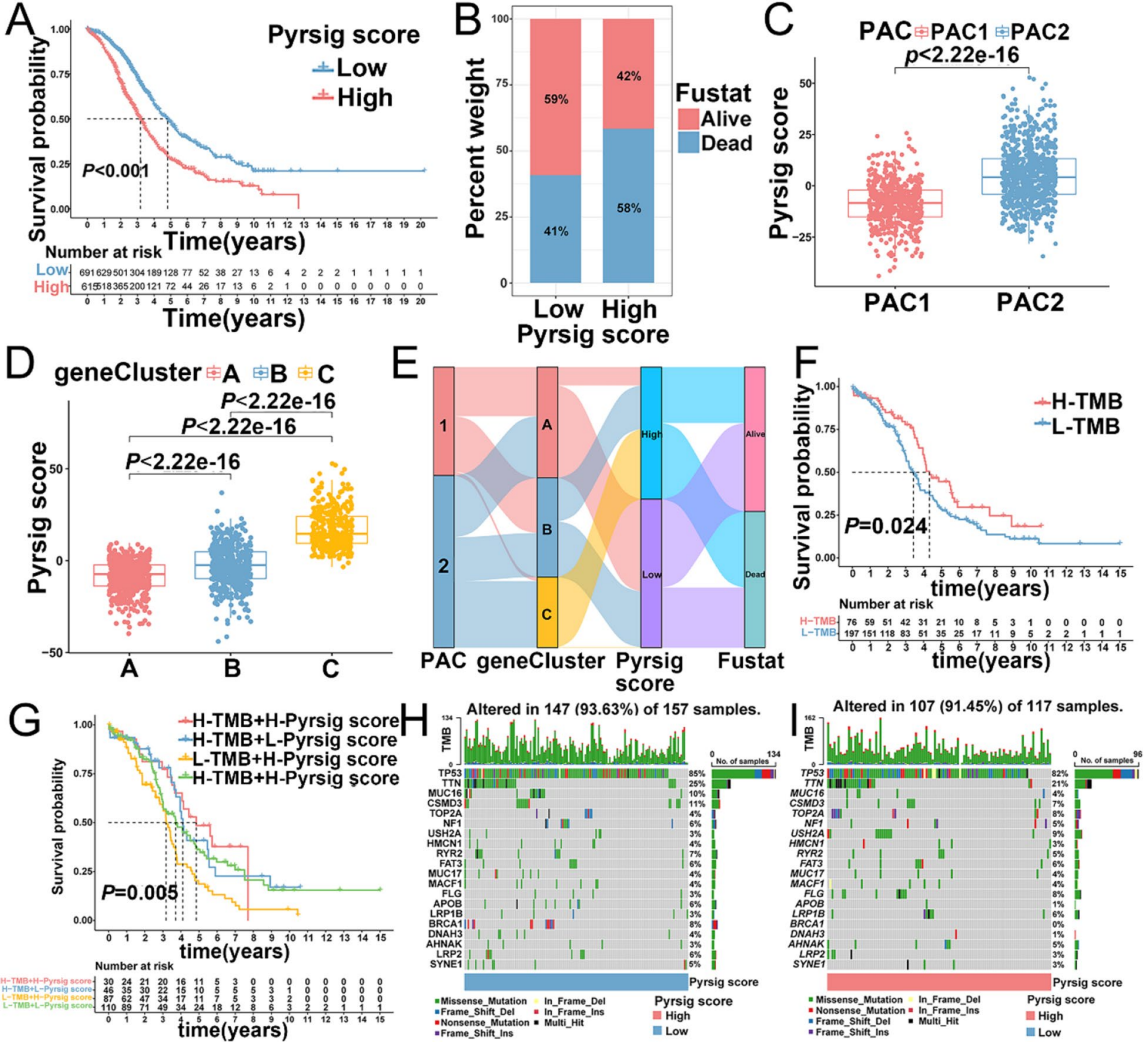
Construction of Pyrsig score and exploring the relationship between Pyrsig score and clinical features.** A**, **B** The relationship between survival outcome and Pyrsig score in patients from TCGA and five GEO cohorts (Log-rank test, *P* < 0.001). **C**,** D** Level of Pyrsig score in two PACs and three gene clusters (Kruskal-Wallis H test, *P* < 0.01). **E** Distributions of two PACs, three gene clusters, Pyrsig scores and survival outcomes in OC from TCGA-OV and five GEO cohorts with Alluvial diagram **F** Survival analysis of patients with low and high TMB in TCGA-OV cohort with Kaplan-Meier. **G** Difference in prognostic advantages among four groups stratified by Pyrsig score and TMB in TCGA-OV cohort shown by Kaplan-Meier curves (Log-rank test, *P* = 0.005). **H**,** I** Somatic mutation features established with low and high Pyrsig scores by waterfall plot. H-TMB: High tumor mutation burden; L-TMB: Low tumor mutation burden

Given that the crucial regulation function of PYAGs in prognostic evaluation and TME immune landscapes, we further constructed a scoring system with PCA algorithm, termed as Pyrsig score, to quantify pyroptosis regulation and immune microenvironment in individual OC sample. We showed that low Pyrsig score (59% alive and 41% dead) presented prominent survival advantage than high Pyrsig score group (42% alive and 58% dead) (*P* < 0.001, Fig. 5A, B), indicating that Pyrsig score showed potentially prognostic value of OC patients. More importantly, significant differences in Pyrsig score were observed among different PACs (Fig. 5C) and three gene clusters (Fig. 5D), gene cluster A showed the lowest Pyrsig score while gene cluster C presented the highest Pyrsig score (Additional file 9: Table S11) (all *P* < 0.001). The distribution of samples in two PACs (PAC1 and PAC2), three gene clusters (gene cluster A/B/C), and two Pyrsig score groups (low and high score) were illustrated with alluvial diagram (Fig. 5E), indicating that gene cluster C with PAC2 displayed a higher Pyrsig score, showing worse survival outcome, whereas gene cluster A with PAC1 exhibited a lower Pyrsig score with favorable prognosis (Fig. 5E). Additionally, Pyrsig score showed no statistic difference between serous ovarian cancer and others types in OC (*P* > 0.05, Additional file 5: Fig. S5A-B) and displayed statistical significance among different grades (G1-2 vs. G3) (*P* < 0.001, Additional file 5: Fig. S5C-D) and FIGO stages (I-II vs. III-IV) (*P* < 0.01, Additional file 5: Fig. S5E-F). Survival status in patients with serous ovarian cancer (Additional file 5: Fig. S5G), G1-2(Additional file 1: Fig. S5I), G3 (Additional file 5: Fig. S5J), stage III-IV (Additional file 5: Fig. S5L) all displayed statistical significance.

Increasing evidence has demonstrated that somatic mutation patterns were associated with responsiveness to immunotherapy [38]. We found no difference in tumor mutation burden (TMB) between the low and high Pyrsig score groups (Additional file 5: Fig. S5M). However, Kaplan-Meier analysis showed that patients in high TMB group showed better survival outcome than those in low TMB group (*P* = 0.024, Fig. 5F). Through combination of Pyrsig scores and TMB, we revealed that in high Pyrsig score group, the survival rate of patients with high TMB was higher than that of patients with low TMB (log-rank test, *P* = 0.005, Fig. 5G). We then analyzed the distribution variations of somatic mutations between the two Pyrsig score groups in TCGA-OV cohort. The top ten mutated genes were TP53, TTN, MUC16, CSMD3, TOP2A, NF1, USH2A, HMCN1, RYR2, and FAT3 (Fig. 5H, I). Patients with a high Pyrsig score showed higher frequencies of USH2A, TOP2A and FLG mutations compared to those with a low Pyrsig score. However, opposite effect was observed regarding the mutation levels of TP53, TTN, and MUC16 (Fig. 5H, I). Moreover, we assessed potential correlation between Pyrsig score and Cancer Stem Cell (CSC) index in OC, and found that CSC index (RNAss and DNAss) showed no significant correlation with Pyrsig score in OC (both *P* > 0.05) (Additional file 5: Fig. S5N-O), indicating that OC cells with different Pyrsig score possessed no distinct stem cell properties and cell differentiation.

## The role of Pyrsig score in TME and immune checkpoints of OC

**Fig. 6:**
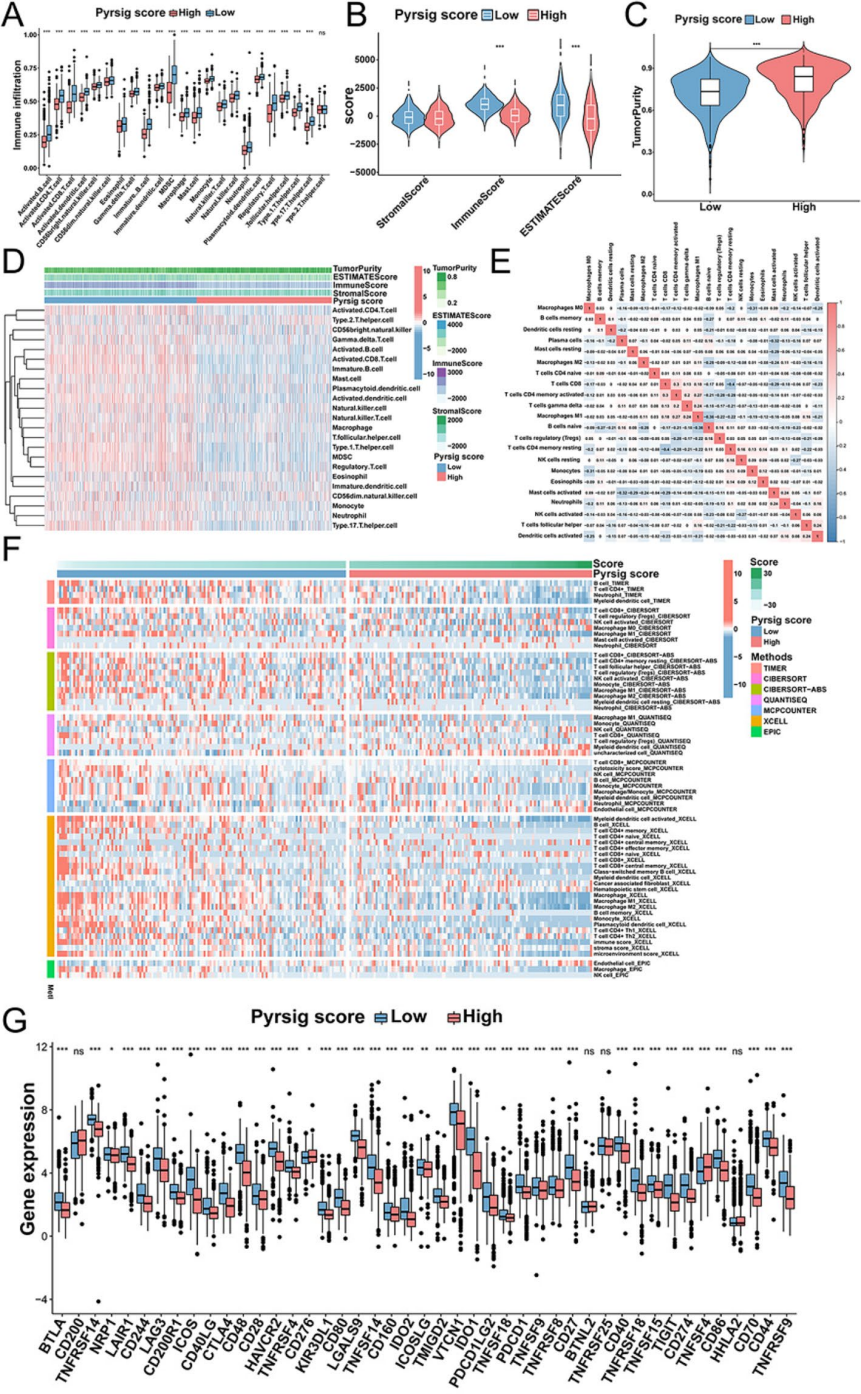
Evaluating the relationship of Pyrsig score with TME and immune-related characteristics.** A** The abundance of 23 tumor-infiltrating immune cells in low and high Pyrsig scores with Estimate algorithm (Kruskal-Wallis H test). **B**,** C** TME score and tumor purity of different Pyrsig scores shown by vioplot. **D** Correlation between TME score, tumor-infiltrating immune cells and Pyrsig score shown by pheatmap. **E** Correlation matrix of all 22 immune cell proportions by heatmaps. **F** Heatmap for TME infiltrating cells and immune score based on TIMER, CIBERSORT, CIBERSORT − ABS, QUANTISEQ, MCP_counter, XCELL and EPIC algorithms in different Pyrsig scores. **G** Differences in the expression levels of immune checkpoints in different Pyrsig scores. *P < 0.05; **P < 0.01; ***P < 0.001

We firstly performed ssGSEA algorithm to evaluate TME immune infiltration in different Pyrsig score groups, and detected that infiltration of immune cells, including activated B cells, activated CD4^+^/CD8^+^ T cells, natural killer cell, eosinophil, mast cell, plasmacytoid dendritic cell, were significantly lower in patients with low Pyrsig score than those with high Pyrsig score (Fig. 6A). We also found negative correlation between Pyrsig score and stromal score (*R*=-0.11, *P* = 0.00013), immune score (*R*=-0.72, *P* < 2.2e-16), ESTIMATE score (*R*=-0.47, *P* < 2.2e-16), and positive correlation between Pyrsig score and tumor purity (*R*=-0.47, *P* < 2.2e-16) (Fig. 6B-D and Additional file 6: Fig. S6A-D), suggesting that low Pyrsig score could predict immune activation-related features. Furthermore, we integrated TIMER, CIBERSORT, CIBERSORT − ABS, QUANTISEQ, MCP_COUNTER, XCELL, and EPIC to calculate the levels of immune cell infiltration in OC samples, which demonstrated that immune cells with dominant immune activation and anti-tumor activity were prominently enriched in low Pyrsig score group, such as CD8^+^ T cell _TIMER, Macrophage M1_XCELL, central memory CD8^+^ T cell _XCELL, plasmacytoid dendritic cell_XCELL, NK cell activated_CIBERSORT-ABS, NK cell_MCPCOUNTER, myeloid dendritic cell activated_XCELL, macrophage M1_XCELL, central memory CD8^+^ T cell _XCELL, (Fig. 6E, F and Additional file 6: Fig. S6E), further confirming the crucial role of pyroptosis in tumor immune infiltration.

Furthermore, we investigated the correlation between immune checkpoints and Pyrsig score, and showed that patients with low Pyrsig score presented significantly higher expression of most of immune checkpoints compared with patients with high Pyrsig score group, including PD-1, PD-L1, CTLA-4, LAG3 and TNFRSF9 (Fig. 6G). Our researches also showed significantly higher expressions of chemokines, interleukins, interferons and MHC complex in low Pyrsig score group (Additional file 6: Fig. S6F-I), and regulators related to TGF-β/EMT pathway (ACAT2, VIM, COL4A1, TGFB2, PDGFRA, TWIST1 and SMAD9) were significantly upregulated in high Pyrsig score group (Additional file 6: Fig. S6J). The above results revealed that Pyrsig score was associated with the immune infiltration of TME in OC.

## Pyrsig score predicts chemotherapy sensitivity and immunotherapy

We comprehensively evaluated the sensitivities of chemotherapy drugs in different Pyrsig score groups, patients in low Pyrsig score group showed higher IC50 value for chemotherapeutics including cisplatin, vinorelbine, docetaxel, and doxorubicin (Additional file 7: Fig. S7A and S7D-F). While IC50 values of paclitaxel, olaparib, gemcitabine, gefitinib, sunitinib were significantly lower in the patients with low Pyrsig score group than those in high Pyrsig score group (Additional file 7: Fig. S7B-C and Additional file 7: Fig. S7G-I), suggesting that Pyrsig score could predicted the sensitivity of chemotherapeutic drug.

**Fig. 7:**
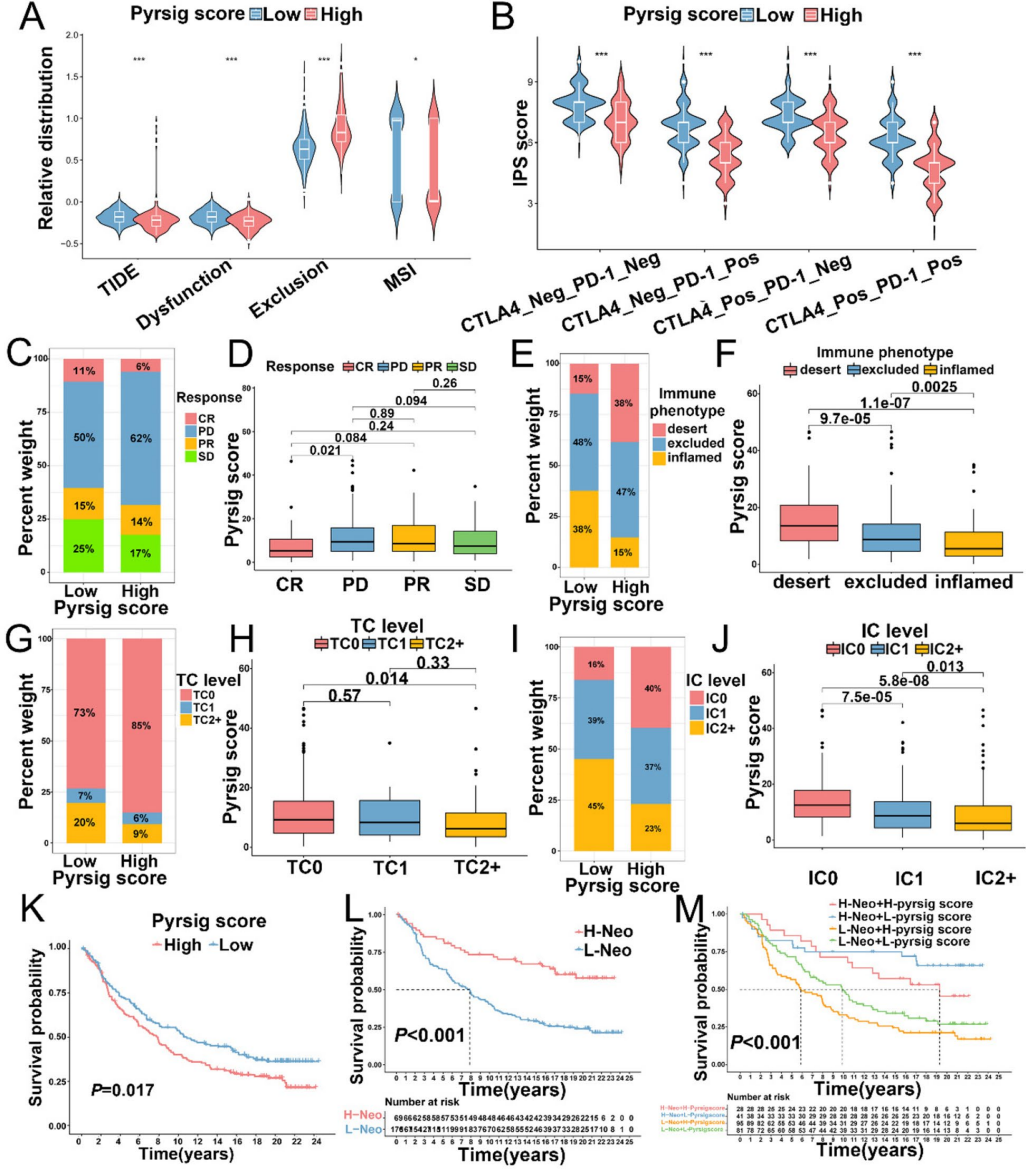
Relationships between Pyrsig score and responsiveness to anti-PD-L1 immunotherapy. **A**, **B** Relative distribution of TIDE **(A)** and IPS **(B)** in different Pyrsig scores in TCGA-OV, respectively. **C, D** The correlation of anti-PD-L1 responsiveness with low and high Pyrsig scores in IMvigor210 database. **E**,** F** The correlation of immune phenotypes with low and high Pyrsig scores in IMvigor210 database. **(G-J)** The correlation of PD-L1 expression on tumor cells **(G**, **H)** and immune cells **(I**, **J)** with low and high Pyrsig scores in IMvigor210 database. **K** OS of patients with different Pyrsig scores in IMvigor210 database by Kaplan-Meier curve. **L** OS of patients with low and high neoantigen burden in IMvigor210 database by Kaplan-Meier curve. **M** OS of patients with anti-PD-L1 immunotherapy stratified by both Pyrsig score and neoantigen burden with Kaplan-Meier curves. SD: stable disease; PD: progressive disease; CR: complete response; PR: partial response. H: high; L: Low; Neo: neoantigen burden

Considering that Pyrsig score was associated with the immune infiltration of tumor microenvironment. Immunotherapies including CTLA-4/PD-L1 inhibitors present a wide developmental foreground in cancer therapy. TIDE and IPS were highly recommended to assess the immune response [26, 36]. We discovered that the TIDE and IPS score were significantly elevated in the low Pyrsig score group (all *P* < 0.001, Fig. 7A, B), which demonstrated Pyrsig score played a crucial role in predicting immune response. Based on the IMvigor210 cohort, we further revealed that patients with CR (complete response) exhibited lowest Pyrsig score than those with PD (partial response) (*P* = 0.21, Fig. 7C, D). The immune infiltration of IMvigor210 cohort was classified into three phenotypes as follows: deserted immune phenotype, inflamed immune phenotype, and excluded immune phenotype. We observed that the Pyrsig score in inflamed immune group was lower than that in the other two groups (*P* < 0.01, Fig. 7E, F). Pyrsig score was also correlated with PD-L1 expression on tumor cells (TC) and immune cells (IC) (Fig. 7G-J). Survival analysis showed that patients with low Pyrsig score exhibited significantly clinical benefits and a markedly prolonged survival following anti-PD-L1 treatment (*P* = 0.017, Fig. 7K), indicating that Pyrsig score could predict responsiveness to anti-PD-L1 immunotherapy. Researches suggested that tumor neoantigen burden played a crucial role in immunotherapeutic efficacy. In IMvigor210 cohort, patients with low neoantigen burden showed poorer clinical outcome than those with high neoantigen burden (*P* < 0.001, Fig. 7L). Furthermore, patients with combination of high neoantigen burden and low Pyrsig score presented a great prognostic advantage (*P* < 0.001, Fig. 7M). The above work strongly indicated that Pyrsig score was significantly correlated with tumor immune characteristic and response to anti-PD-L1 immunotherapy, and the established Pyrsig score would contribute to predicting prognosis of patients.

### Development of a nomogram to predict survival of OC

**Fig. 8:**
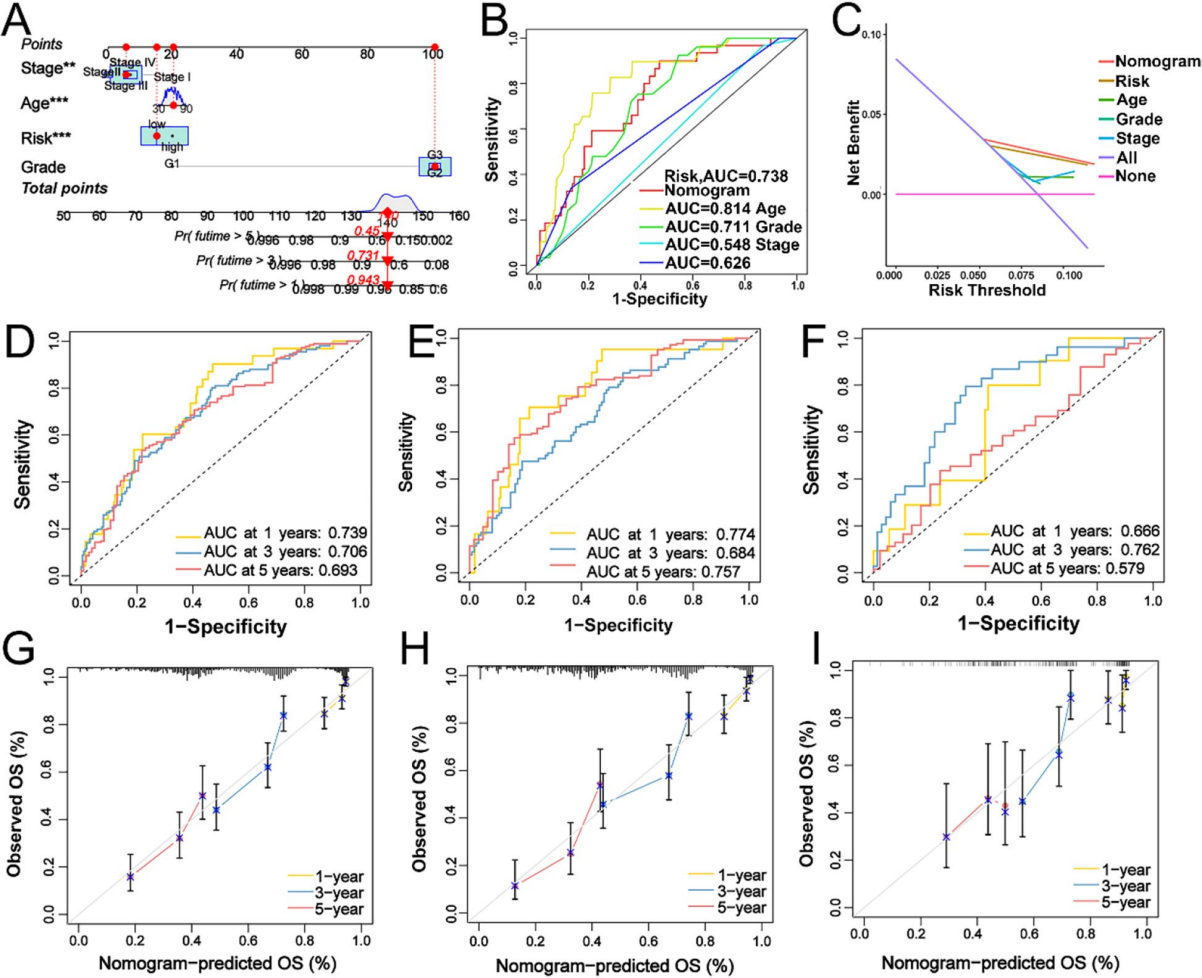
Construction and validation of Pyrsig score as an independent prognosis factor in a nomogram.** A** Nomogram for predicting the probability of patient mortality at 1-, 3- and 5- year OS of OC patients based on four independent prognosis factors. **B** ROC curves of Pyrsig score and clinical parameters of OC in TCGA-OV cohort. **C** DCA curves of Pyrsig score and clinical parameters of OC in TCGA-OV cohort. **D-F** ROC curves for predicting the 1-, 3-, and 5- year OS of OC patients in all TCGA, training sets and testing sets. **G-I** Calibration curves of the nomogram for predicting of 1-, 3-, and 5- year OS of OC patients in all TCGA, training and testing sets. ROC, receiver operating characteristic. DCA, decision curve analysis. OS: overall survival. **P < 0.01, ***P < 0.001

Considered the inconvenience clinical utility of Pyrsig score in predicting OS in patients with OC, a nomogram integrating Pyrsig score and clinicopathological parameters (age, FIGO stage and differentiation grade) was constructed to predict 1-, 3-, and 5- year OS (Fig. 8A). Time-dependent ROC analysis demonstrated the nomogram exhibited much more powerful capacity of survival prediction compared with other clinicopathological characteristics, with an average AUC above 0.814 (Fig. 8B). Pyrsig score suggested the most powerful capacity for survival prediction and clinical parameters of OC in TCGA cohort with DCA curves (Fig. 8C). AUC experiments on the nomogram model showed higher accuracy for OS at 1-, 3-, and 5- years in the whole TCGA, training set, testing set (Fig. 8D-F). In the calibration analysis, the prediction lines of the nomogram for 1-, 3-, and 5- year survival probability were extremely close to the ideal performance (Fig. 8G-I), indicating a high accuracy of the nomogram and powerful capacity for prognostic prediction.

### Validation of the expression levels of GSDMD and GZMB in ovarian cancer

**Fig. 9:**
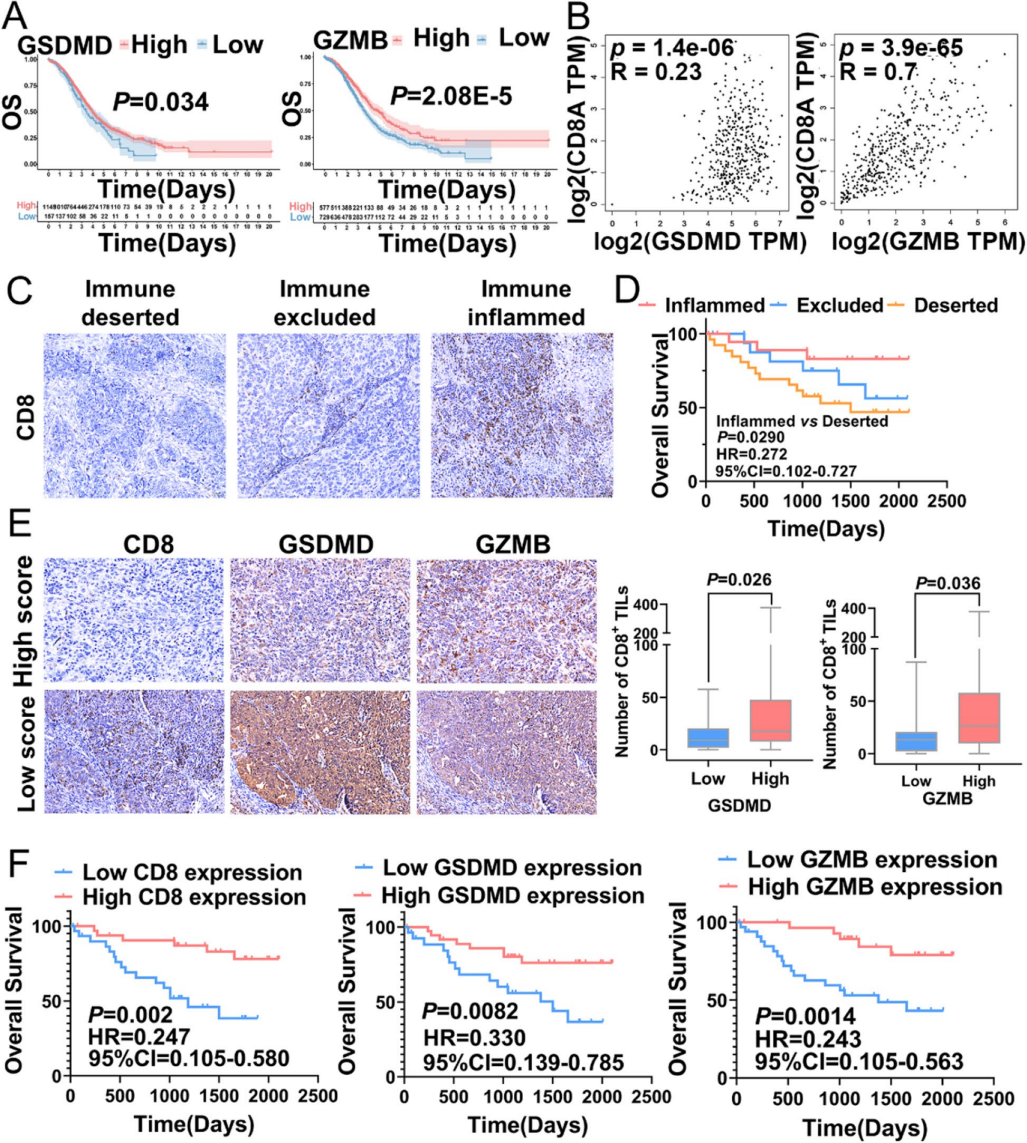
Validation of the expression levels of GSDMD and GZMB in ovarian cancer.** A** The OS between high and low GSDMD and GZMB in TCGA-OV and five GEO databases shown by Kaplan-Meier curve. **B** The correlation between GSDMD, GZMB and CD8 expression in TCGA-OV samples with Spearman analysis. **C** Expression of CD8 in three immune subtypes of ovarian cancer microarray cohort detected by immunohistochemistry (scale bar: 50 μm). **D** Differences in overall survival of three immune subtypes of ovarian cancer microarray cohort shown by Kaplan-Meier curve. **E**, **F** Expressions of CD8, GZMB and GSDMD in ovarian cancer microarray cohort detected by immunohistochemistry (scale bar: 50 μm). **G** Validation of the prognostic value of CD8, GZMB and GSDMD in ovarian cancer samples

As shown in Additional file 2: Fig. S2H, GZMB was a protective factor in OC, we further explored the expression of 49 PYAGs in different Pyrsig score group and found that expressions of GSDMD and GZMB were significantly higher in low Pyrsig score group than those in high Pyrsig score group (Additional file 8: Fig. S8A). We further found that high expressions of GSDMD and GZMB displayed favorable prognosis in OC from six cohorts (*P* = 0.034 and *P* = 2.08E-5, respectively) (Fig. 9A), GSDMD and GZMB were significantly associated with the expression of immune checkpoint molecules with pan-cancer analysis (Additional file 8: Fig. S8B-C). We further investigated the relationship of two genes with CD8 expression, and found that expression of CD8 were positively correlated with GSDMD (*R* = 0.23, *P*-value = 1.4e-06) and GZMB (*R* = 0.7, *P-*value = 3.9e-65) (Fig. 9B). Furthermore, according to the expression of CD8 in tissues microarray with IHC, OC samples were classified into three immune types: immune desert/excluded/inflamed tumors (Fig. 9C), and the inflammed subtype showed prolonged survival than deserted subtype (HR = 0.272, 95%CI = 0.102–0.727, *P* = 0.029) (Fig. 9D). IHC showed that patients with higher infiltration of CD8^+^ T cells which indicated low Pyrsig score group, showed higher expression GSDMD and GZMB (*P* = 0.026 and *P* = 0.036, respectively) and lymphatic metastasis (*P* = 0.046) (Fig. 9E-F and Additional file 9: Table S12). The expression of GSDMD and GZMB were both significantly correlated with Pyrsig score (*P* = 0.018 and *P* = 0.034, respectively) in 65 ovarian cancer patients (Additional file 9: Table S13). Kaplan-Meier analysis showed high expression of CD8, GSDMD and GZMB exhibited better survival outcome (Fig. 9G). We further employed COX regression model to explore clinicopathological parameters affecting prognosis of ovarian cancer with forest heatmap. Univariate analysis showed GZMB expression (HR = 0.218, 95%CI = 0.081–0.59, *P* = 0.023), GSDMD expression (HR = 0.378, 95%CI = 0.163–0.875, *P* = 0.003), CD8 expression (HR = 0.229, 95%CI = 0.089–0.588, *P* = 0.002) and age at diagnosis (HR = 2.78, 95%CI = 1.032–7.496, *P* = 0.043) were significantly associated with overall survival of OC (Additional file 8: Fig. S8E). Multivariate analysis showed that GZMB expression (HR = 0.296, 95%CI = 0.1-0.876, *P* = 0.028) and CD8 expression (HR = 0.321, 95%CI = 0.122–0.848, *P* = 0.022) were charactered as independent prognostic factors of OC (Additional file 8: Fig. S8F). As such, these findings confirms that GSDMD and GZMB plays a crucial role in mediating immune infiltration.

## Discussion

Mounting evidence demonstrated that pyroptosis took on an indispensable role in inflammation, immune response as well as antitumor effect through interaction with a variety of components in TME [39, 40]. The landscape of TME infiltration characterizations mediated by integrated effects of multiple PYAGs have not yet been comprehensively recognized in ovarian cancer. Here, we firstly revealed universal alterations in PYAGs at the transcriptional and genetic heterogeneity in OC, and found that different expression of PYAGs may be associated with regulation of genome variation. It has now been widely recognized that methylation modifications were involved in various cancer and antitumor effect of chemotherapy and immunotherapy. Therefore, we performed methylation analysis of 51 PYAGs and showed that AIM2, CASP1, CASP8, GSDMC and NLRP6 exhibited hypomethylation in ovarian cancer, which may provide a new theory to explore the methylation of above five genes to improve potential antitumor efficacy of OC. Then we identified two distinct PACs based on the expression of PYAGs in OC, showing that PAC1 was characterized by immune fully-activated biological behaviors and pathways, including natural killer cell mediated cytotoxicity, cytokine-cytokine receptor interaction, T and B cell receptor signaling pathway, Toll-like and NOD-like receptor signaling pathways, corresponding to an immune-inflamed phenotype. While PAC2 was mainly charactered by immune suppression biological process, corresponding to an immune-desert phenotype. We also identified PAC1 was significantly associated with elevated activated immune cells and higher immune score, which confirmed the reliability of our classification of immune phenotypes for different PACs. Therefore, PAC1 presented immune activated characteristics and survival advantage, contributing to potential predictive value on immunotherapy advantages.

Furthermore, based on the prognostic significance and immune regulation of DEGs between two PACs, which were regarded as pyroptosis-related gene signatures, and then three pyroptosis gene subtypes were established and showed profound potential on prognostic prediction and immunotherapy response of OC, further playing a crucial role in shaping different landscapes of TME. Therefore, a comprehensive evaluation of pyroptosis subtypes will facilitate our understanding of TME cell-infiltrating characterization. Moreover, we established a trusty and effective Pyrsig scoring system to evaluate pyroptosis of individual patients with OC, PAC2 and gene cluster C exhibited a higher Pyrsig score with poor survival, respectively. Subsequent studies highlighted that Pyrsig score could serve as a prognostic predictor in OC and significantly associated with different clinicopathological characteristics, immune infiltration of TME, immune checkpoints, chemotherapeutic drug susceptibility and immunotherapy, indicating a predictive advantage in precision immunotherapy for ovarian cancer, which can be employed for prognosis stratification of patients with OC, deepening our understanding of pyroptosis molecular mechanism and providing new ideas for targeted therapies.

We also revealed the relative abundance of 22 immune cells and immune-related molecules displayed significant differences in the two PACs (PAC1 and PAC2), three gene clusters (A/B/C) and different Pyrsig score groups. Patients in PAC1 and low Pyrsig score group, with survival advantage, exhibited higher infiltration of M1 macrophages, activated memory CD4^+^ and CD8^+^ T cells, Gamma delta T cells, and activated NK cells. Amounting studies revealed that tumor-associated macrophages (TAM) were classified into M1 macrophages (promoting antitumor immunity) or M2 macrophages (boosting tumor progression) [41]. M1 macrophage polarization in TME of OC played a tumoricidal role and was correlated with prolonged survival [42]. Infiltration of M2 macrophages could promote tumor cell migration and invasion [43], and exert the function of inhibiting immunity, predicting a worse survival outcome [44]. In accordance with previous publications, we observed that the abundance of M1 macrophages in PAC1 and low Pyrsig score group were higher than those in PAC2 and high Pyrsig score group. Increasing evidence has showed that activated T cells played a crucial role in anti-tumor immunity of OC [45, 46]. High gamma delta T cell infiltration upon stimulation could suppress tumor progression via multiple mechanisms in OC [47]. Furthermore, activated NK cells exhibited multiple functions on combating immune escape and directly and indirectly target cells clearance [48]. Patients in PAC1 and low Pyrsig score group showed higher infiltration of activated memory CD4^+^ and CD8^+^ T cells, gamma delta T cells and activated NK cells, suggesting a better prognosis and anti-tumor immunity in OC development, we also validated GSDMD and GZMB were significantly associated infiltration of CD8^+^ T cells in OC with IHC. Infiltration of Tregs, as the potential immunosuppressive cells in immune system, promoted tumor progression through dampening antitumor immunity and boosting angiogenic reprogramming of TME [49]. This were in line with our findings of higher Tregs infiltration in the TME of patients with PAC2 and high Pyrsig score group, which showed poor survival outcome.

The combination of chemotherapy with immunotherapy, such as immune checkpoint blockade, demonstrates profound clinical application value for tumor treatment [50]. We showed significant differences in the efficacy of chemotherapeutic drug of different Pyrsig score groups, indicating that Pyrsig score could guide the precision usage of chemotherapeutic drugs. Immunotherapies with immune checkpoint inhibitors, such as PD-1, PD-L1, and CTLA-4 inhibitors, have demonstrated promising survival advantages in metastatic melanoma, metastatic renal cancer, and non-small cell lung cancer for the past few years [51–53]. In consideration of these developments, we observed that patients with a low Pyrsig score showed better response with anti-PD-L1 immunotherapy, indicating that Pyrsig score could predict clinical outcome of patients with immune checkpoint blockade application. Our researches laid a foundation for a deeper understanding of patients’ antitumor immune response and provided guidance for more individualized and effective immunotherapy regimen with novel Pyrsig score.

However, there still existed several limitations of our research. First, all data and samples involved in this study were collected from public databases retrospectively. although we have avoided batch effect to the great extent, it still had some influence, which may have affected the efficacy and survival outcome of immunotherapy. Furthermore, numerous prospective researches and additional experimental validations in *vivo* and *vitro* are still required to further confirm the underlying mechanisms of PYAGs.

## Conclusion

In conclusion, we comprehensively explored genetic variations and transcriptional patterns of pyroptosis in 1332 OC samples, and uncovered their potential role on prognostic value, clinicopathological characteristics and tumor immune microenvironment of ovarian cancer, we further revealed the superior advantage of Pyrsig score in targeted therapy and immunotherapy, and expressions of GSDMD and GZMB were associated with immune infiltration. These results highlighted that evaluating the pyroptosis subtypes of the individual tumor will contribute to enhancing our cognition of TME infiltration characteristics and yielded novel insights into personalized and effective immunotherapeutic strategies.

## Supplementary Information

**Additional file 1:** **Figure S1.** The workflow of our work.

**Additional file 2:** **Figure S2.** Relationships between TP53 mutation, DNA methylation, survival outcomes and PYAGs in patientswith OC.

**Additional file 3:** **Figure S4.** Characteristics of chemokines, interleukins, interferons, and other cytokines among the threedistinct pyroptosis gene clusters.

**Additional file 4:** **Figure S4.** Characteristics of chemokines, interleukins, interferons, and other cytokines among the threedistinct pyroptosis gene clusters.

**Additional file 5:** **Figure S5.** Comprehensive analysis of Pyrsig score in OC.

**Additional file 6:** **Figure S6.** Tumor immune infiltrationcharacteristics and expression levels of chemokines, interleukins, interferons,and other cytokines between low and high Pyrsig score groups.

**Additional file 7:** **Figure S7.** Relationshipsbetween Pyrsig score and chemotherapeutic sensitivity.

**Additional file 8:** **Figure S8.** Relationships between immune checkpoints and GSDMD, GZMB and their prognostic values.

**Additional file 9:** **Table S1.** Basic characteristics of datasets enrolled in this study for identifying pyroptosis-associated genes (PYAGs) signatures. **Table S2.** Demographic and clinical characteristics of 1334 ovarian cancer patients from TCGA-OV and  five GEO cohorts. **Table S3.** Summary of 51 recognized  PYAGs. **Table S4.** Summary of DNA methylation modifications of 51  PYAGs with DiseaseMeth 2.0. **Table S5.** The prognostic values of 49 PYAGs in OC patients of TCGA-OV and  five GEO cohorts.** Table S6.** Spearman correlation analysis of the 49  PYAGs in OC. **Table S7.** The activation states of biological pathways in distinct pyroptosis-associated clusters (PACs) by GSVA enrichment analysis. **Table S8.** The TME score and tumor purity of OC samples were analyzed with ESTIMATETable S9. Functional annotation of the differentially expressed genes（DEGs） between the two PACs.. Table S9. Functional annotation of the differentially expressed genes（DEGs） between the two PACs.Table S9. Functional annotation of the differentially expressed genes（DEGs） between the two PACs. **Table S9.** Functional annotation of the differentially expressed genes（DEGs） between the two PACs. **Table S10.** Prognostic analysis of 889 DEGs using a univariate Cox analysis. **Table S11.** Distribution of PYAG scores in the different pyroptosis subtypes (Kruskal-Wallis H test, *P*<0.01).  **Table S12.** Relationships between the expressions of CD8, GSDMD, GZMB and clinicopathological parameters of 65 ovarian cancer patients. **Table S13.** Correlation between Pyrsig score and expressions of GSDMD, GZMB in 65 ovarian cancer patients.

## Data Availability

All data generated or analyzed during this study are collected from the Cancer Genome Atlas (TCGA, https://portal.gdc.cancer.gov/), and Gene Expression Omnibus (GEO, https://www.ncbi.nlm.nih.gov/geo/).

## Abbreviations

OC: Ovarian cancer
CNV: Copy number variation
TMB: Tumor mutation burden
TCGA: The Cancer Genome Atlas
GEO: Gene-Expression Omnibus
TME: Tumor microenvironment
PCA: Principal component analysis
GSVA: Gene set variation analysis
ssGSEA: Single-sample gene-set enrichment analysis
DEGs: Differentially expressed genes
EMT: Epithelial-mesenchymal transition
OS: Overall survival
IHC: Immunohistochemistry
