# Supplementary figures and images for: Identification and validation of pyroptosis-related gene landscape in prognosis and immunotherapy of ovarian cancer
Source: J Ovarian Res. 2023 Jan 27;16:27. doi: 10.1186/s13048-022-01065-2 (PMC9883900; doi:10.1186/s13048-022-01065-2)

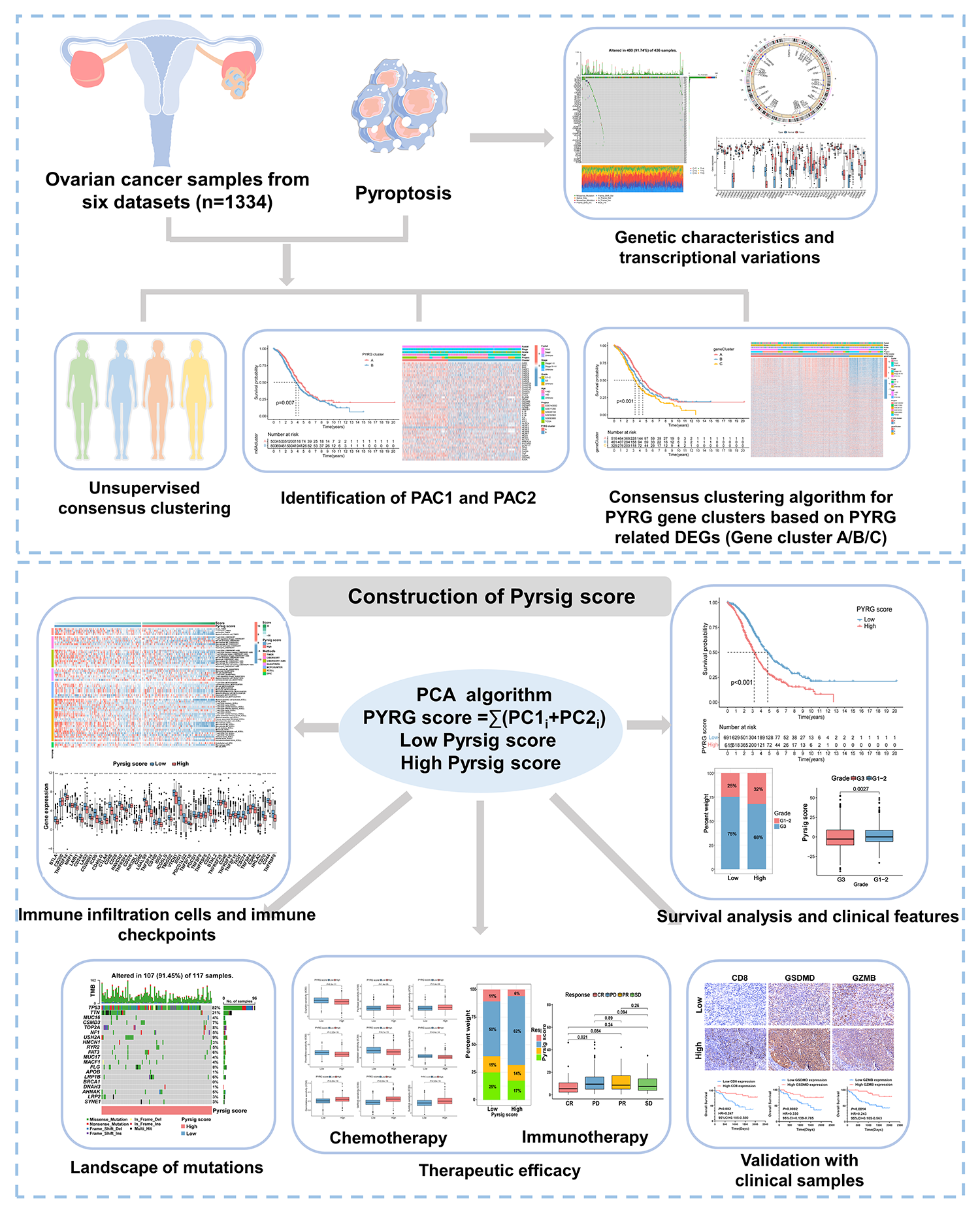


**Supplementary Figure S1. The workflow of our work**

Supplement: Supplementary file 1 — Additional file 1: Figure S1. The workflow of our work. [file 13048_2022_1065_MOESM1_ESM.doc]
