# Supplementary material for: Identification and validation of pyroptosis-related gene landscape in prognosis and immunotherapy of ovarian cancer
Source: J Ovarian Res. 2023 Jan 27;16:27. doi: 10.1186/s13048-022-01065-2 (PMC9883900; doi:10.1186/s13048-022-01065-2)
Supplement: Supplementary file 2 — Additional file 2: Figure S2. Relationships between TP53 mutation, DNA methylation, survival outcomes and PYAGs in patientswith OC. [file 13048_2022_1065_MOESM2_ESM.doc]

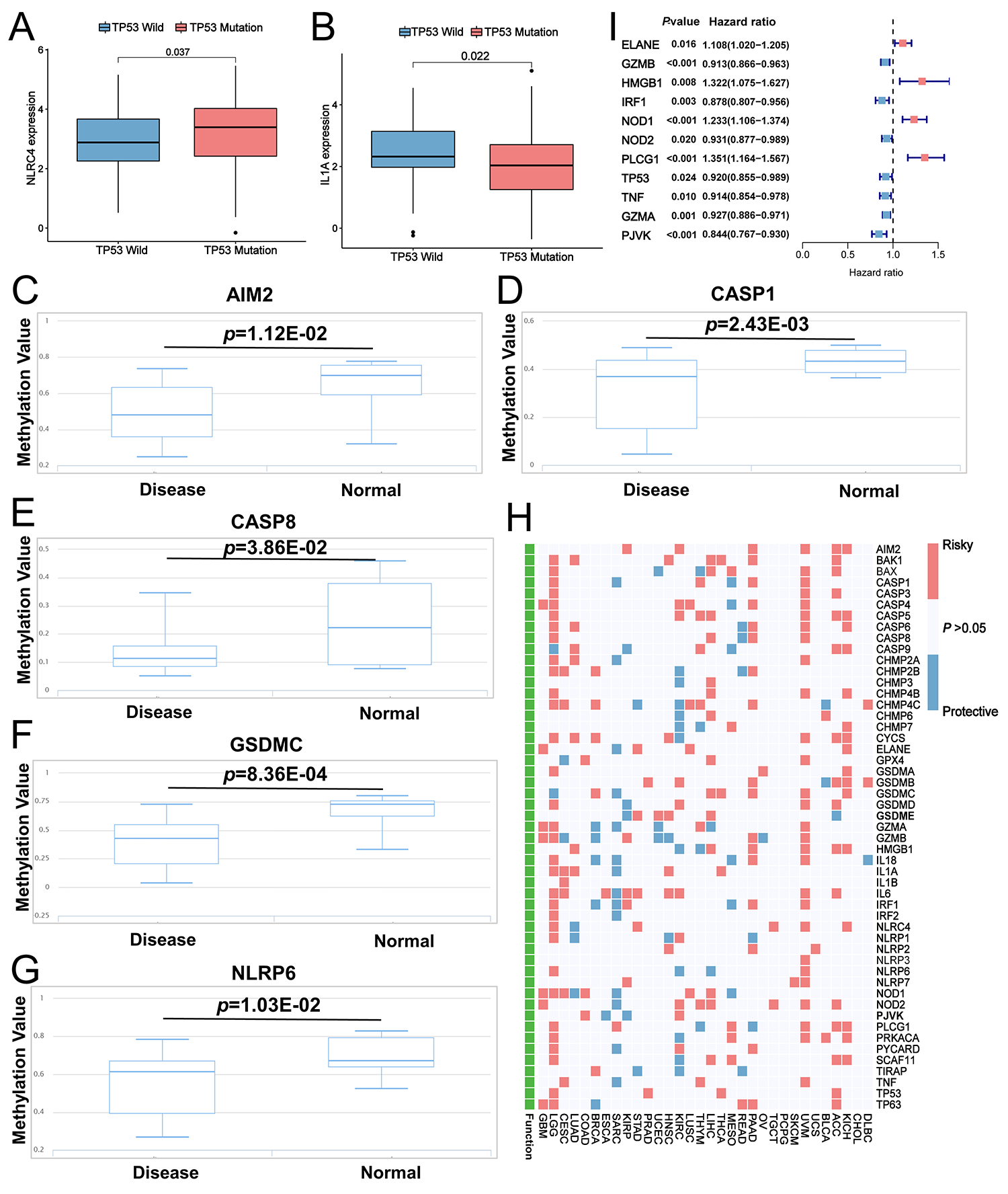


**Supplementary Figure S2. Relationships between TP53 mutation, DNA methylation, survival outcomes and PYAGs in patients with OC. (A-B)** Difference in two PYAGs expressions (NLRC4 and IL1A) between TP53-mutant and wild types in ovarian cancer. **(C-G)** DNA methylation levels of AIM2, CASP1, CASP8, GSDMC and NLRP6 analyzed with DiseaseMeth 2.0. **(H)** Summary of the correlation between expression of PYAGs and survival outcomes across 33 cancer type. Red represents higher expression of PYAGs associated with worse survival, and blue represents higher expression of PYAGs associated with better survival. Only P values < 0.05 were shown. **(I)** Prognostic analysis for PYAGs in TCGA-OV and five GEO cohorts with a Univariate Cox regression model. PYAGs: pyroptosis-associated genes.

.
