# Supplementary material for: Identification and validation of pyroptosis-related gene landscape in prognosis and immunotherapy of ovarian cancer
Source: J Ovarian Res. 2023 Jan 27;16:27. doi: 10.1186/s13048-022-01065-2 (PMC9883900; doi:10.1186/s13048-022-01065-2)
Supplement: Supplementary file 3 — Additional file 3: Figure S4. Characteristics of chemokines, interleukins, interferons, and other cytokines among the threedistinct pyroptosis gene clusters. [file 13048_2022_1065_MOESM3_ESM.doc]

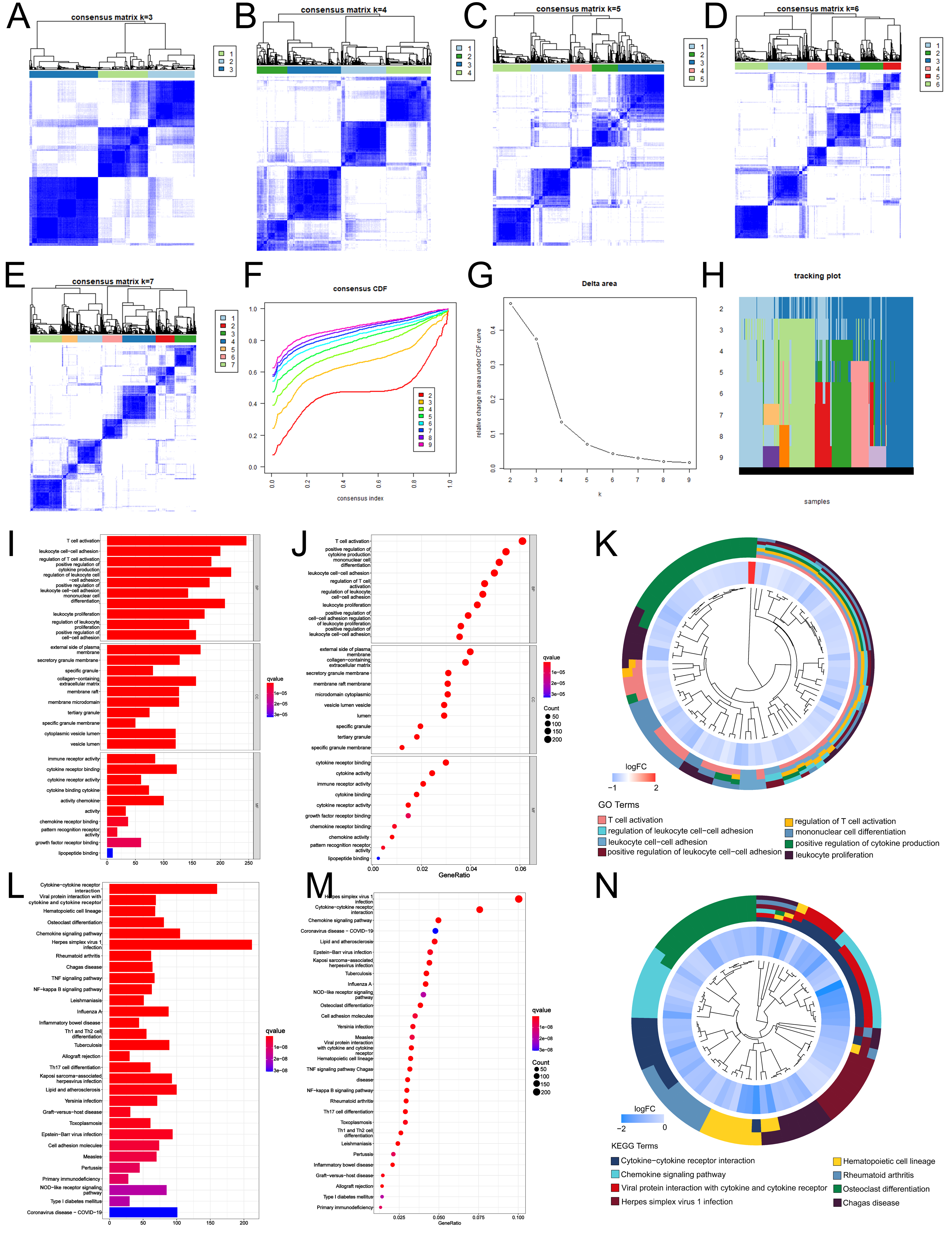


**Supplementary Figure S3 GO and KEGG enrichment analysis of differentiated expressed genes (DEGs) between the two distinct PYAG clusters. (A-H)** Unsupervised clustering of PYAGs and consensus matrix heatmaps for *k* = 3-7. **(I-K)** GO enrichment analysis of biological characteristics of DEGs between two PACs. **(L-N)** KEGG enrichment analysis of DEGs between two PACs. GO: Gene Ontology; KEGG: Kyoto Encyclopedia of Genes and Genomes.


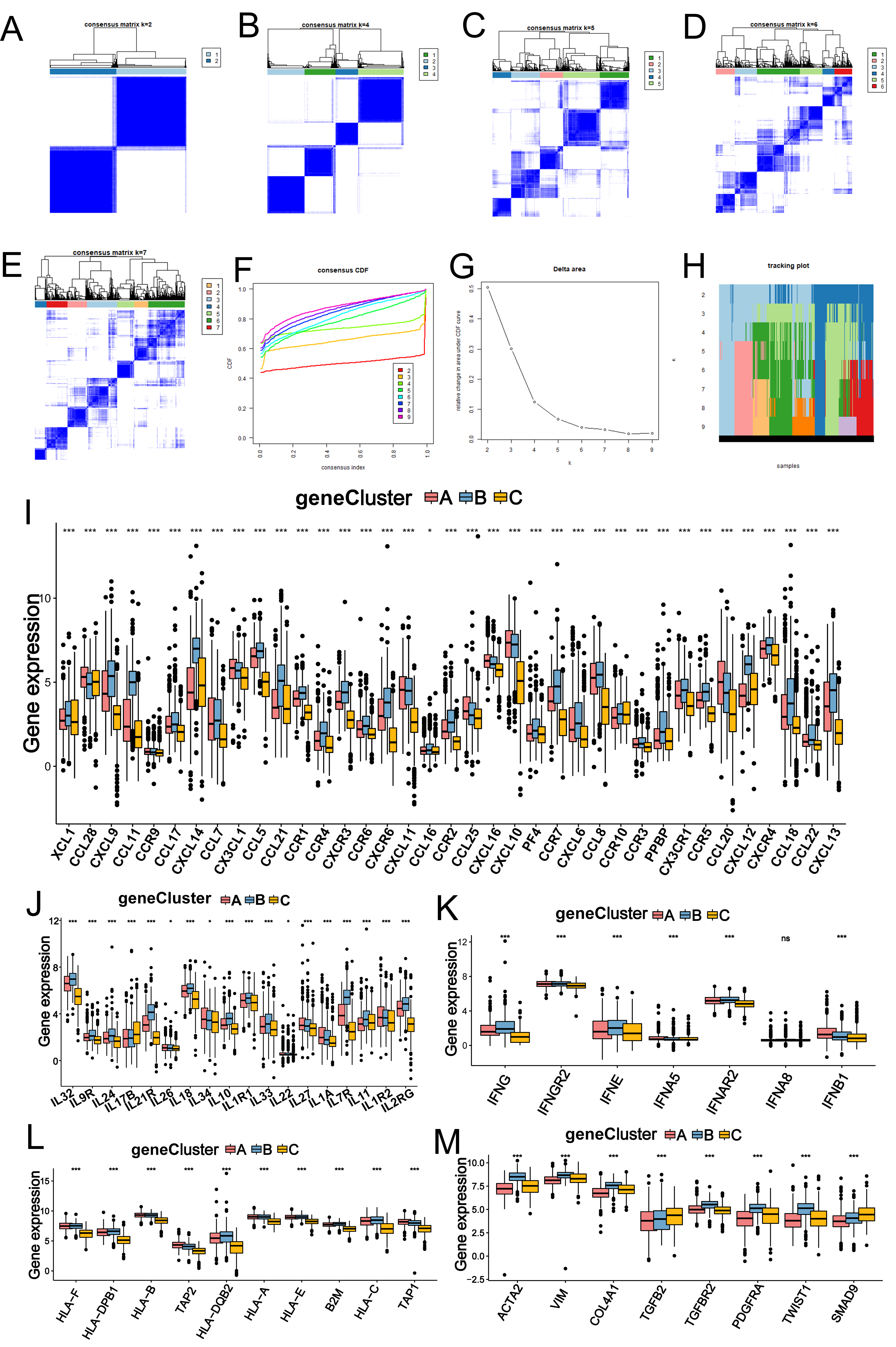


**Supplementary Figure S4 Characteristics of chemokines, interleukins, interferons, and other cytokines among the three distinct pyroptosis gene clusters.** **(A-H)** Unsupervised clustering of 889 DEGs related to pyroptosis and consensus matrixes for *k* = 2 and 4-7. **(I-L)** Difference in expressions of chemokines, interleukins, interferons and MHC molecules among three gene clusters. **(M)** Difference in expressions of TGF-β/EMT pathway-related genes among three gene clusters. **P* < 0.05; ***P* < 0.01; ****P* < 0.001.


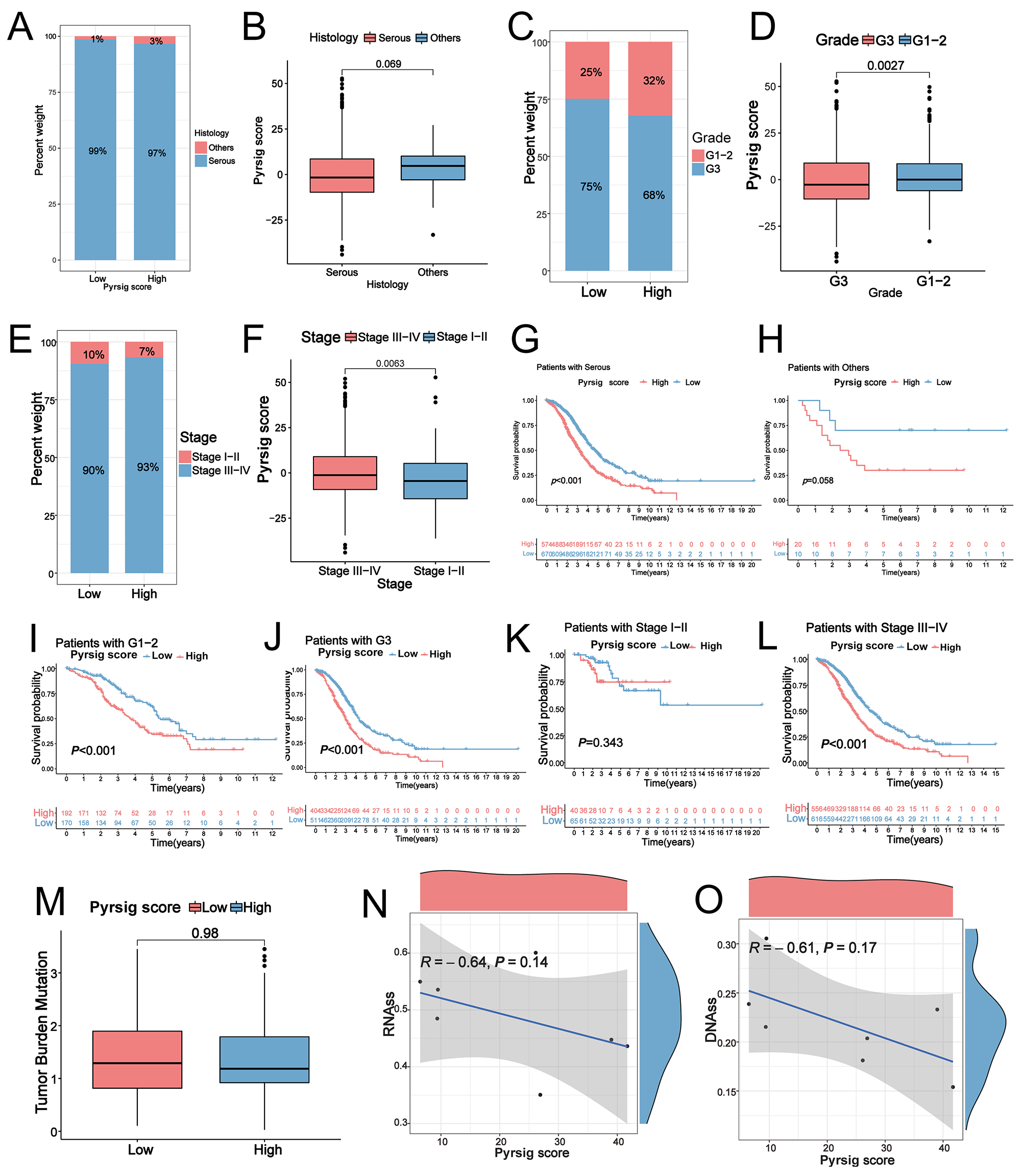


**Supplementary Figure S5 Comprehensive analysis of Pyrsig score in OC. (A-B)** Relationships between Pyrsig score and different histology (serous vs others) in OC. **(C-D)** Relationships between Pyrsig score and tumor grade (G1-2 vs G3) in OC. **(E-F)** Relationships between Pyrsig score and FIGO stages (I-II vs III-IV) in OC. **(G-H)** Survival analysis of Pyrsig scores in OC patients with serous ovarian cancer and other types by Kaplan-Meier. **(I-J)** Survival analysis of Pyrsig scores in OC patients with G1-2 and G3 by Kaplan-Meier. **(K-L)** Survival analysis of Pyrsig score in OC patients with Stage I-II and Stage III-IV by Kaplan-Meier. **(M)** Relationships between Pyrsig score and tumor burden mutation in OC. **(N-O)** Relationships between Pyrsig score and CSC index in OC. CSC: Cancer Stem Cell.


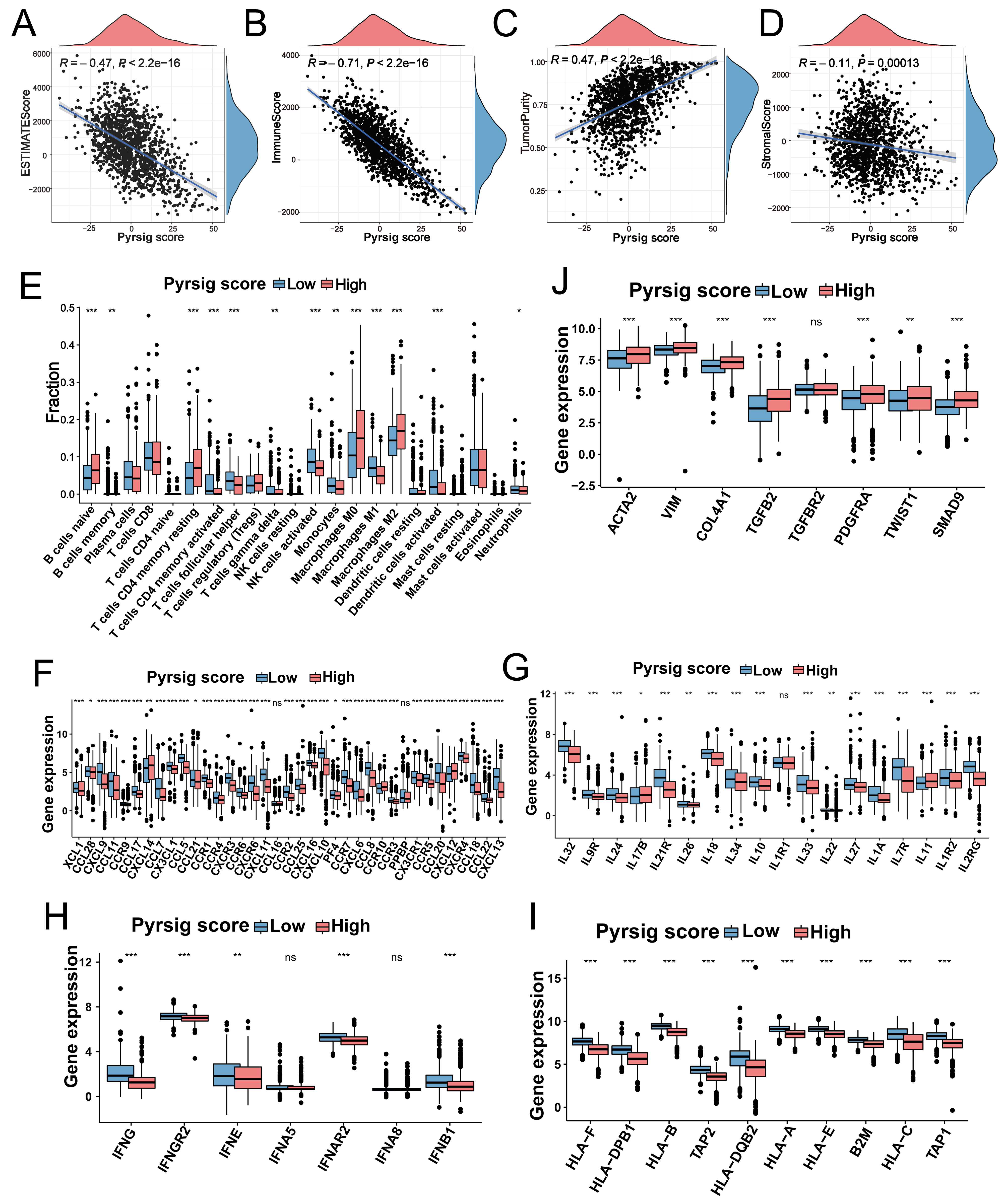


**Supplementary Figure S6 Tumor immune infiltration characteristics and expression levels of chemokines, interleukins, interferons, and other cytokines between low and high Pyrsig score groups. (A-D)** Correlation between TME score, tumor purity and Pyrsig score in OC. **(E)** Fraction of tumor-infiltrating immune cells in two Pyrsig score groups detected by the CIBERSORT algorithm. **(F-I)** Difference in expressions of chemokines, interleukins, interferons and MHC molecules between low and high Pyrsig score groups. **(J)** Difference in expressions of TGF-β/EMT pathway-related genes between low and high Pyrsig score groups. **P*<0.05; ***P*<0.01; ****P*<0.001.


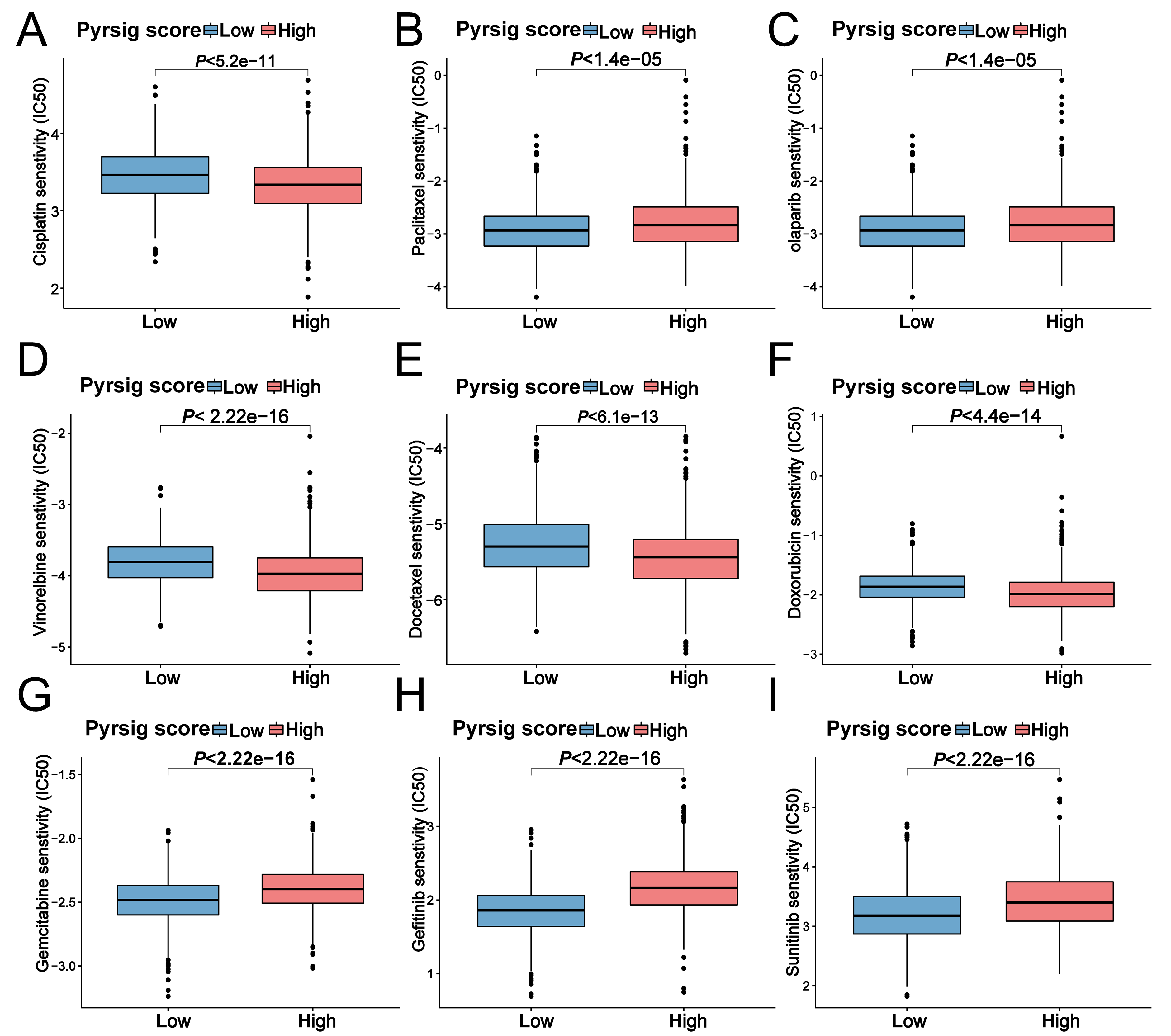


**Supplementary Figure S7 Relationships between Pyrsig score and chemotherapeutic sensitivity. (A-I)** IC50 value of Cisplatin **(A)**, Paclitaxel **(B)**, Olaparib **(C)**, Vinorelbine **(D)**, Docetaxel **(E)**, Doxorubicin **(F)**, Gemcitabine **(G)**, Gefitinib **(H)**, Sunitinib **(I)** in low and high Pyrsig score groups (all *P*<0.05).


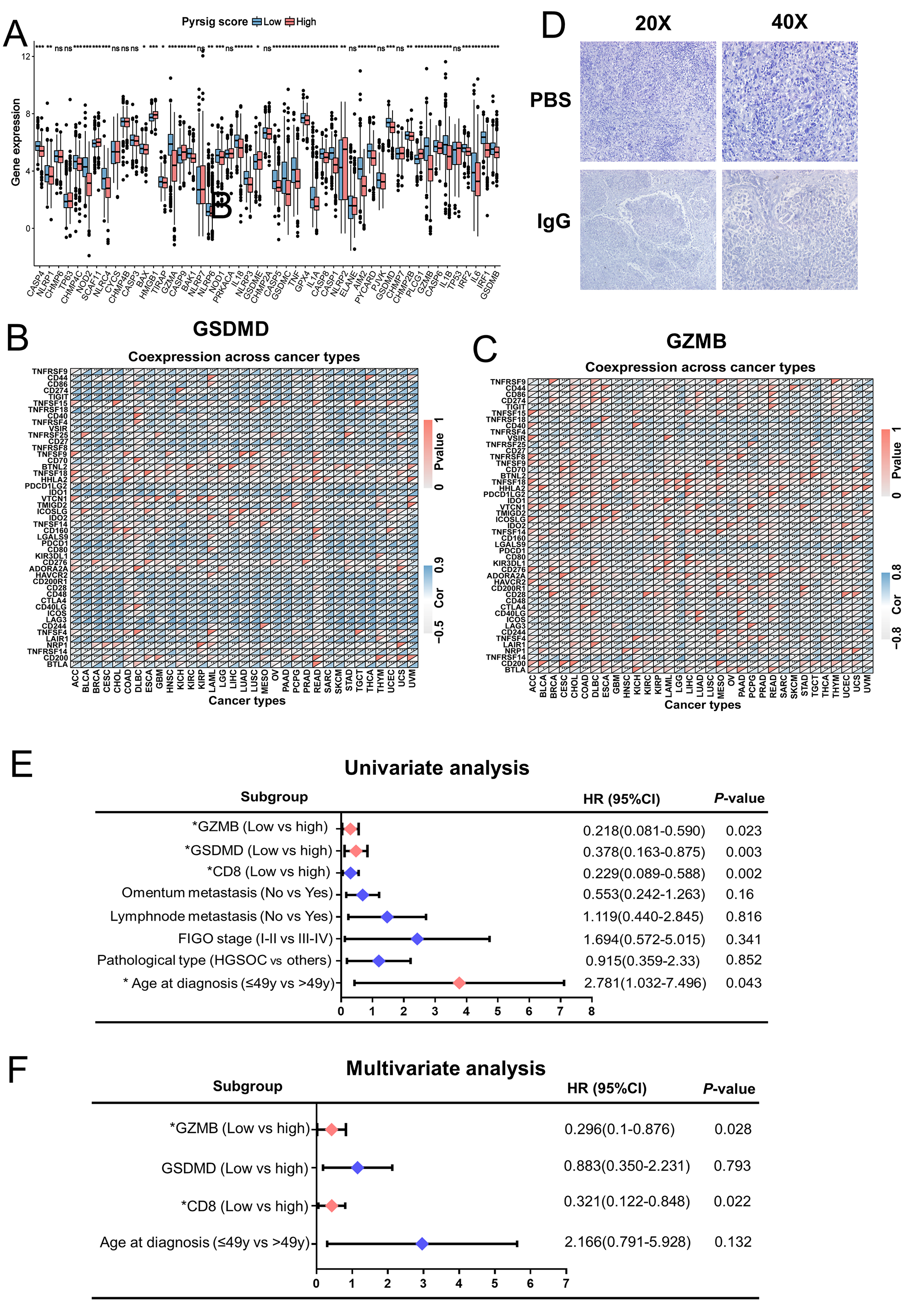


**Supplementary Figure S8 Relationships between immune checkpoints and GSDMD, GZMB and their prognostic values. (A-B)** The correlation between GSDMD, GZMB and immune checkpoint molecules with pan-cancer analysis. **(C)** The negative and isotype control with PBS and Rabbit IgG detected by IHC, respectively. **(D-E)** Univariate **(D)** and multivariate **(E)** Cox regression analysis by forest map.
