# Supplementary material for: Identification and validation of pyroptosis-related gene landscape in prognosis and immunotherapy of ovarian cancer
Source: J Ovarian Res. 2023 Jan 27;16:27. doi: 10.1186/s13048-022-01065-2 (PMC9883900; doi:10.1186/s13048-022-01065-2)
Supplement: Supplementary file 4 — Additional file 4: Figure S4. Characteristics of chemokines, interleukins, interferons, and other cytokines among the threedistinct pyroptosis gene clusters. [file 13048_2022_1065_MOESM4_ESM.doc]

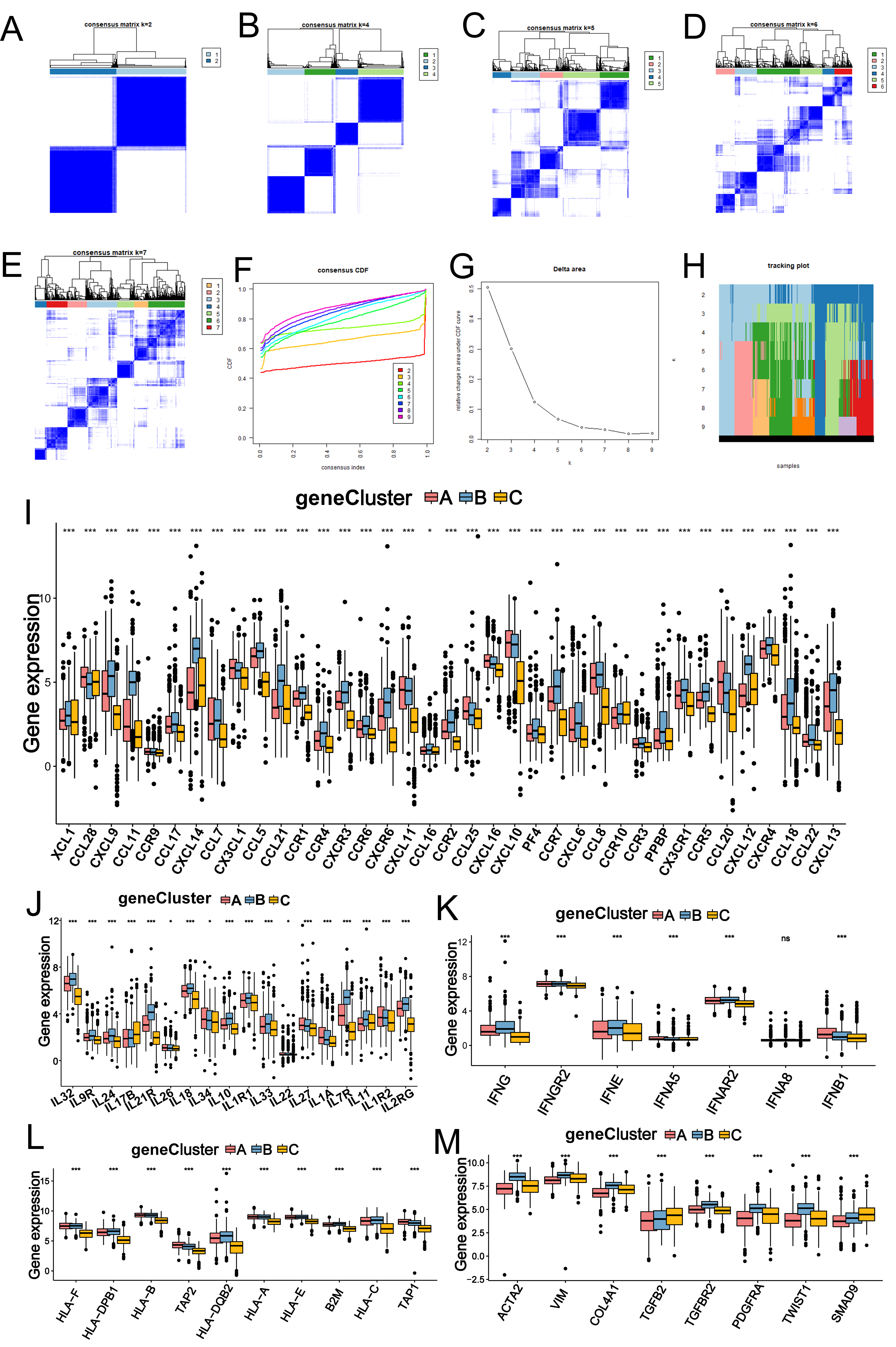


**Supplementary Figure S4. Characteristics of chemokines, interleukins, interferons, and other cytokines among the three distinct pyroptosis gene clusters.** **(A-H)** Unsupervised clustering of 889 DEGs related to pyroptosis and consensus matrixes for *k* = 2 and 4-7. **(I-L)** Difference in expressions of chemokines, interleukins, interferons and MHC molecules among three gene clusters. **(M)** Difference in expressions of TGF-β/EMT pathway-related genes among three gene clusters. **P* < 0.05; ***P* < 0.01; ****P* < 0.001.
