# Supplementary material for: Identification and validation of pyroptosis-related gene landscape in prognosis and immunotherapy of ovarian cancer
Source: J Ovarian Res. 2023 Jan 27;16:27. doi: 10.1186/s13048-022-01065-2 (PMC9883900; doi:10.1186/s13048-022-01065-2)
Supplement: Supplementary file 5 — Additional file 5: Figure S5. Comprehensive analysis of Pyrsig score in OC. [file 13048_2022_1065_MOESM5_ESM.doc]

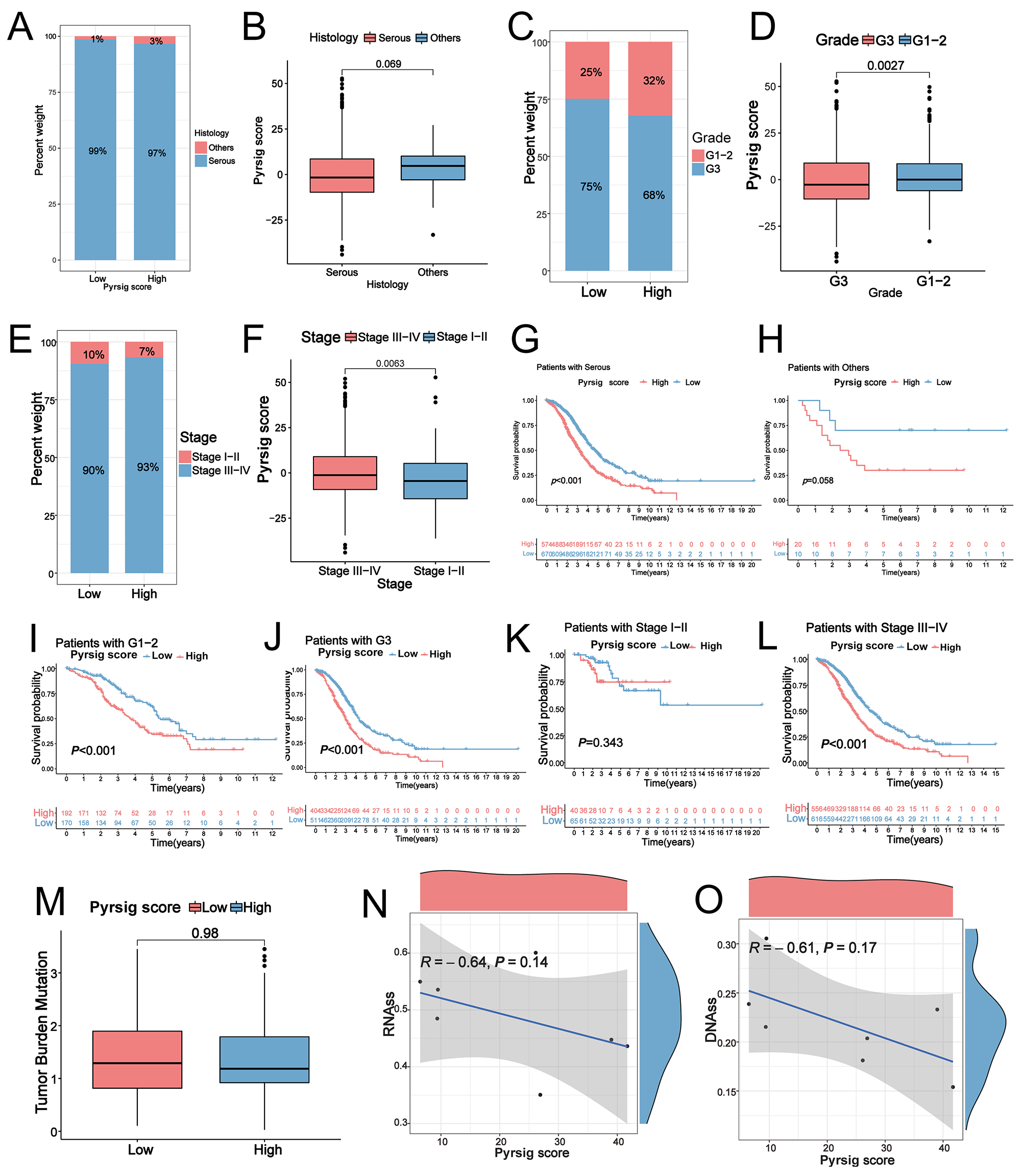


**Supplementary Figure S5. Comprehensive analysis of Pyrsig score in OC. (A-B)** Relationships between Pyrsig score and different histology (serous vs others) in OC. **(C-D)** Relationships between Pyrsig score and tumor grade (G1-2 vs G3) in OC. **(E-F)** Relationships between Pyrsig score and FIGO stages (I-II vs III-IV) in OC. **(G-H)** Survival analysis of Pyrsig scores in OC patients with serous ovarian cancer and other types by Kaplan-Meier. **(I-J)** Survival analysis of Pyrsig scores in OC patients with G1-2 and G3 by Kaplan-Meier. **(K-L)** Survival analysis of Pyrsig score in OC patients with Stage I-II and Stage III-IV by Kaplan-Meier. **(M)** Relationships between Pyrsig score and tumor burden mutation in OC. **(N-O)** Relationships between Pyrsig score and CSC index in OC. CSC: Cancer Stem Cell.
