# Supplementary material for: Identification and validation of pyroptosis-related gene landscape in prognosis and immunotherapy of ovarian cancer
Source: J Ovarian Res. 2023 Jan 27;16:27. doi: 10.1186/s13048-022-01065-2 (PMC9883900; doi:10.1186/s13048-022-01065-2)
Supplement: Supplementary file 6 — Additional file 6: Figure S6. Tumor immune infiltrationcharacteristics and expression levels of chemokines, interleukins, interferons,and other cytokines between low and high Pyrsig score groups. [file 13048_2022_1065_MOESM6_ESM.doc]

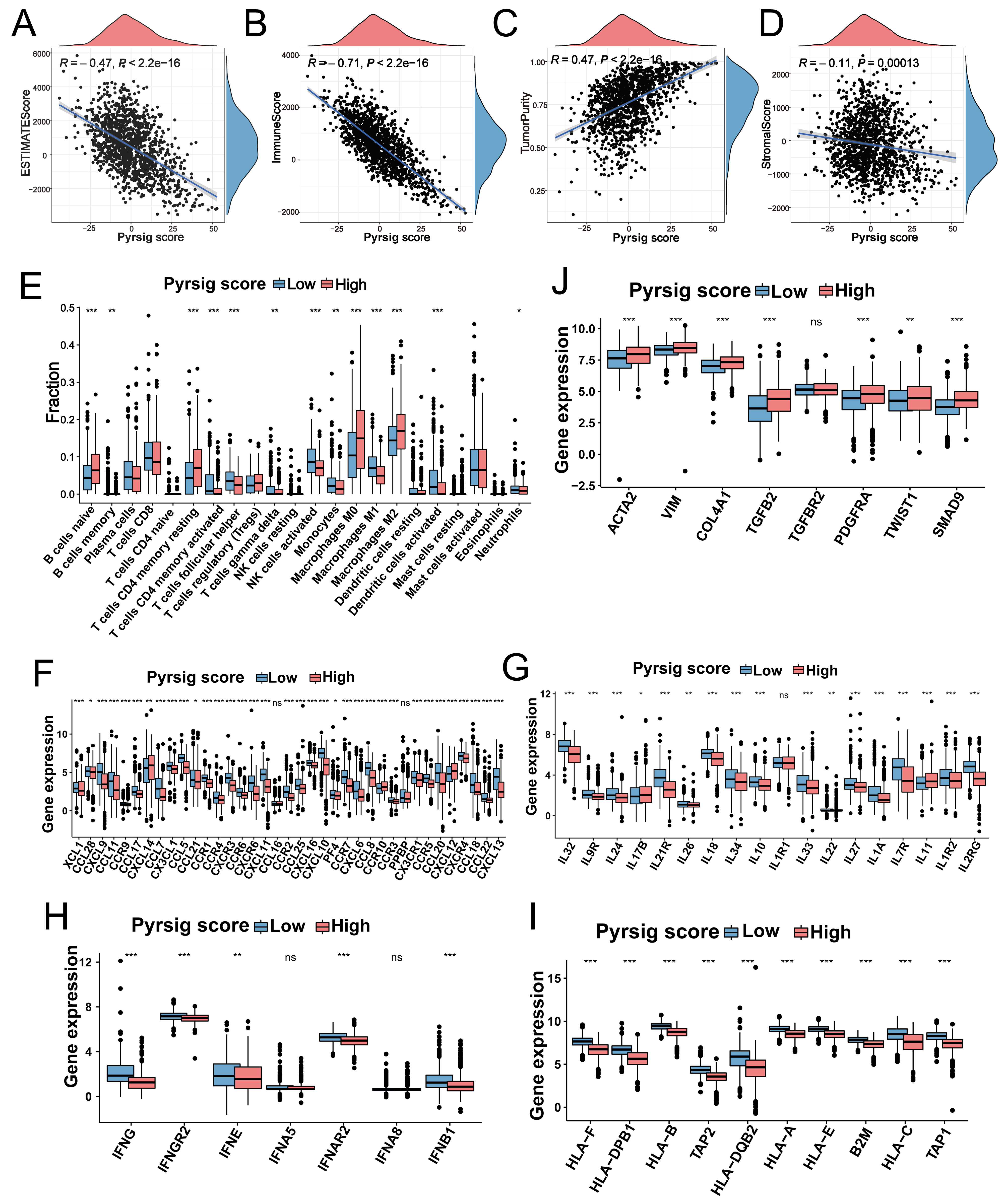


**Supplementary Figure S6 Tumor immune infiltration characteristics and expression levels of chemokines, interleukins, interferons, and other cytokines between low and high Pyrsig score groups. (A-D)** Correlation between TME score, tumor purity and Pyrsig score in OC. **(E)** Fraction of tumor-infiltrating immune cells in two Pyrsig score groups detected by the CIBERSORT algorithm. **(F-I)** Difference in expressions of chemokines, interleukins, interferons and MHC molecules between low and high Pyrsig score groups. **(J)** Difference in expressions of TGF-β/EMT pathway-related genes between low and high Pyrsig score groups. **P*<0.05; ***P*<0.01; ****P*<0.001.
