# Supplementary material for: Identification and validation of pyroptosis-related gene landscape in prognosis and immunotherapy of ovarian cancer
Source: J Ovarian Res. 2023 Jan 27;16:27. doi: 10.1186/s13048-022-01065-2 (PMC9883900; doi:10.1186/s13048-022-01065-2)
Supplement: Supplementary file 7 — Additional file 7: Figure S7. Relationshipsbetween Pyrsig score and chemotherapeutic sensitivity. [file 13048_2022_1065_MOESM7_ESM.doc]

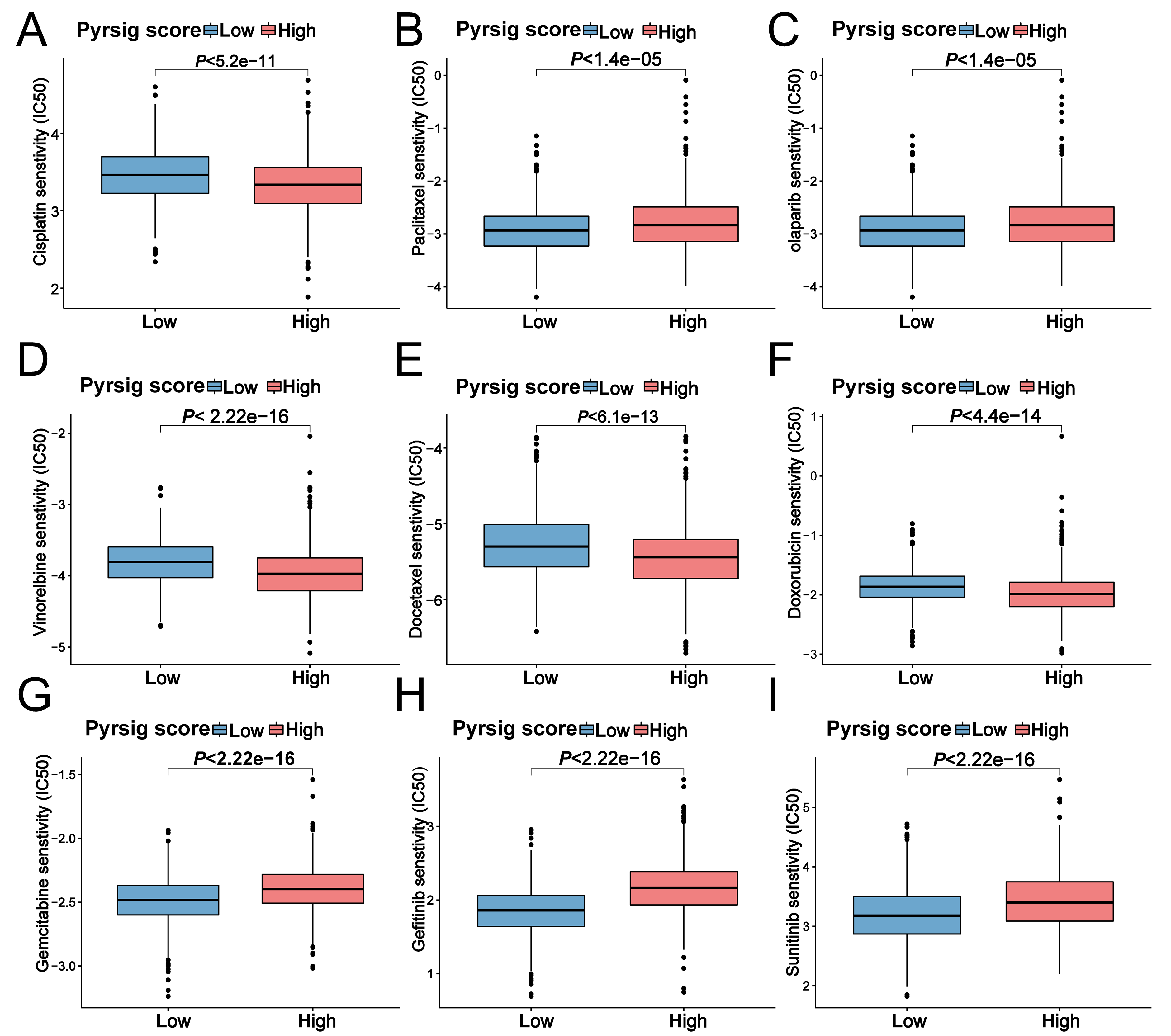


**Supplementary Figure S7. Relationships between Pyrsig score and chemotherapeutic sensitivity. (A-I)** IC50 value of Cisplatin **(A)**, Paclitaxel **(B)**, Olaparib **(C)**, Vinorelbine **(D)**, Docetaxel **(E)**, Doxorubicin **(F)**, Gemcitabine **(G)**, Gefitinib **(H)**, Sunitinib **(I)** in low and high Pyrsig score groups (all *P*<0.05).
