# Supplementary material for: Identification and validation of pyroptosis-related gene landscape in prognosis and immunotherapy of ovarian cancer
Source: J Ovarian Res. 2023 Jan 27;16:27. doi: 10.1186/s13048-022-01065-2 (PMC9883900; doi:10.1186/s13048-022-01065-2)
Supplement: Supplementary file 8 — Additional file 8: Figure S8. Relationships between immune checkpoints and GSDMD, GZMB and their prognostic values. [file 13048_2022_1065_MOESM8_ESM.doc]

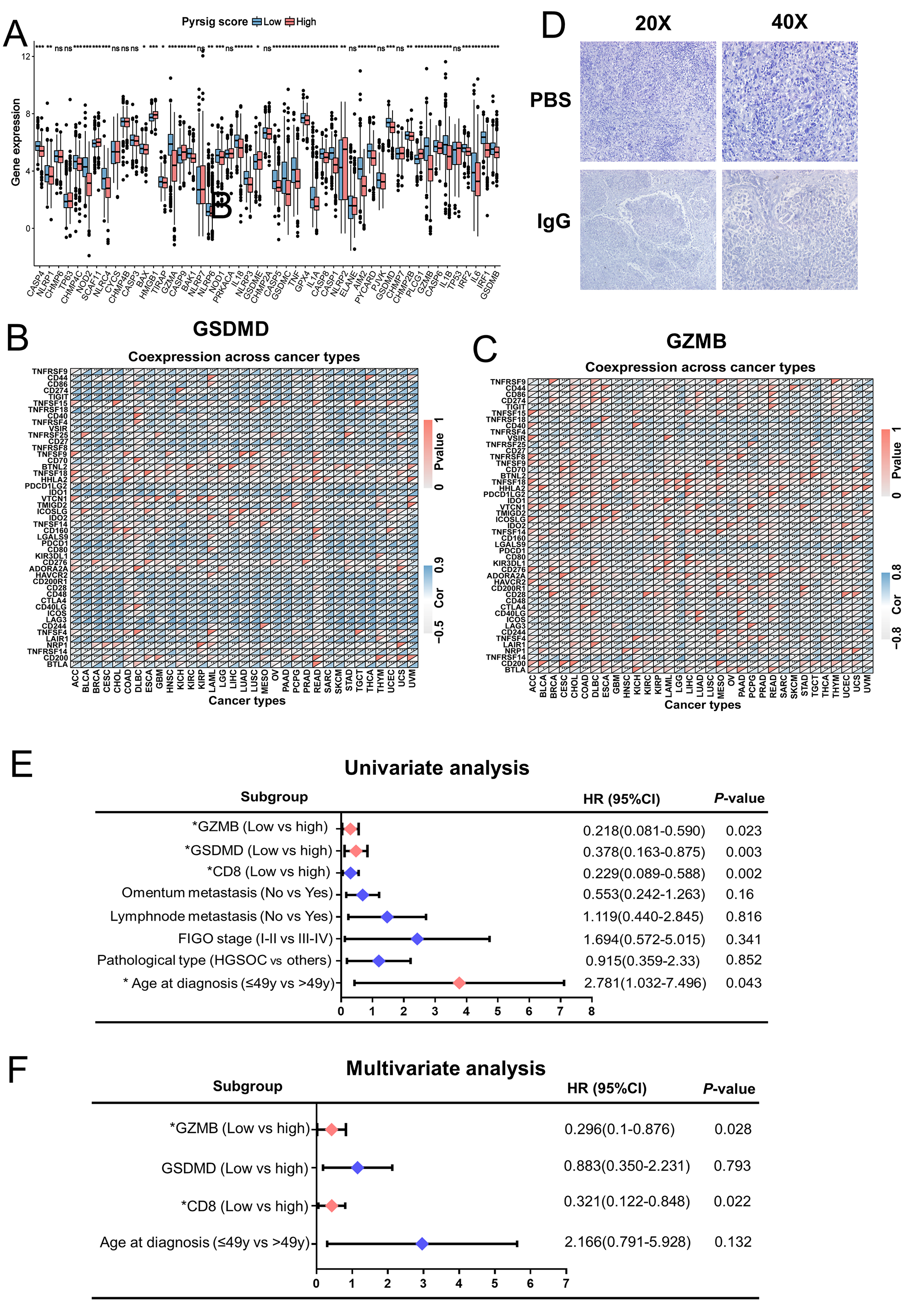


**Supplementary Figure S8. Relationships between immune checkpoints and GSDMD, GZMB and their prognostic values. (A-B)** The correlation between GSDMD, GZMB and immune checkpoint molecules with pan-cancer analysis. **(C)** The negative and isotype control with PBS and Rabbit IgG detected by IHC, respectively. **(D-E)** Univariate **(D)** and multivariate **(E)** Cox regression analysis by forest map.
